# Normalization by distributional resampling of high throughput single-cell RNA-sequencing data

**DOI:** 10.1093/bioinformatics/btab450

**Published:** 2021-06-19

**Authors:** Jared Brown, Zijian Ni, Chitrasen Mohanty, Rhonda Bacher, Christina Kendziorski

**Affiliations:** Department of Statistics, University of Wisconsin Madison, Madison, WI 53706, USA; Department of Statistics, University of Wisconsin Madison, Madison, WI 53706, USA; Department of Biostatistics and Medical Informatics, University of Wisconsin Madison, Madison, WI 53792, USA; Department of Biostatistics, University of Florida Gainesville, Gainesville, FL 32603, USA; Department of Biostatistics and Medical Informatics, University of Wisconsin Madison, Madison, WI 53792, USA

## Abstract

**Motivation:**

Normalization to remove technical or experimental artifacts is critical in the analysis of single-cell RNA-sequencing experiments, even those for which unique molecular identifiers are available. The majority of methods for normalizing single-cell RNA-sequencing data adjust average expression for library size (LS), allowing the variance and other properties of the gene-specific expression distribution to be non-constant in LS. This often results in reduced power and increased false discoveries in downstream analyses, a problem which is exacerbated by the high proportion of zeros present in most datasets.

**Results:**

To address this, we present Dino, a normalization method based on a flexible negative-binomial mixture model of gene expression. As demonstrated in both simulated and case study datasets, by normalizing the entire gene expression distribution, Dino is robust to shallow sequencing, sample heterogeneity and varying zero proportions, leading to improved performance in downstream analyses in a number of settings.

**Availability and implementation:**

The R package, Dino, is available on GitHub at https://github.com/JBrownBiostat/Dino. The Dino package is further archived and freely available on Zenodo at https://doi.org/10.5281/zenodo.4897558.

**Supplementary information:**

Supplementary data are available at *Bioinformatics* online.

## 1 Introduction

Over the past decade, advances in single-cell RNA-sequencing (scRNA-seq) technologies have significantly increased the sensitivity and specificity with which cellular transcriptional dynamics can be analyzed (Bacher and Kendziorski, 2016; Haque *et al.*, 2017; Hwang *et al.*, 2018; Kolodziejczyk *et al.*, 2015; Wu *et al.*, 2017). Further, parallel increases in the number cells which can be simultaneously sequenced have allowed for novel analysis pipelines including the description of transcriptional trajectories (Qiu *et al.*, 2017; Trapnell *et al.*, 2014) and the discovery of rare sub-populations of cells (Hwang *et al.*, 2018; Satija *et al.*, 2015; Tsoucas and Yuan, 2018). The development of droplet-based, unique-molecular-identifier (UMI) protocols such as Drop-seq, inDrop and the 10x Genomics Chromium platform (Klein *et al.*, 2015; Macosko *et al.*, 2015; Zheng *et al.*, 2017) have significantly contributed to these advances. In particular, the commercially available 10x Genomics platform has allowed the rapid and cost-effective gene expression profiling of hundreds to tens of thousands of cells across many studies to date.

**Fig. 1.**
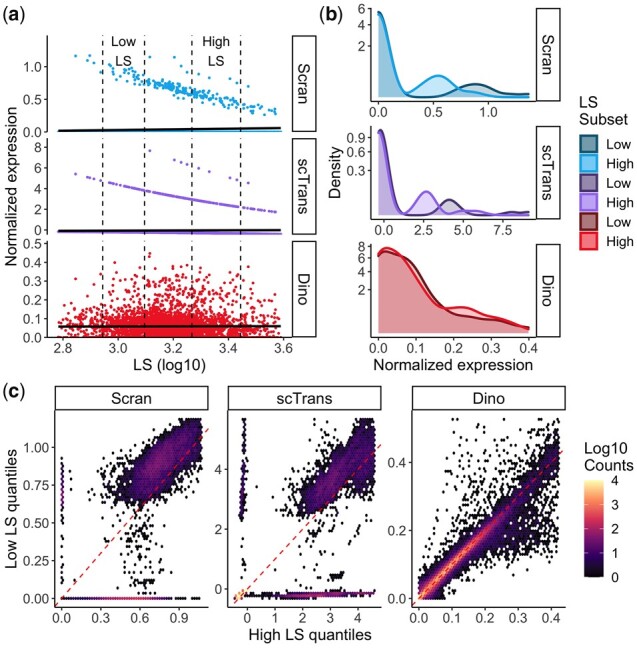
Evaluation of gene-specific expression distributions following normalization. Expression data in the PBMC68K_Pure dataset were normalized by Scran, scTransform and Dino. (**a**) and (**b**) Normalized expression is shown here for a homogeneous set of cells (CD4+/CD45RO+ memory cells) to minimize the effects of cell subpopulation heterogeneity. (a) Normalized expression from a typical gene (NME1) under Scran, scTransform and Dino plotted against LS. Fitted regression lines (solid black) show generally constant means across methods. Low-LS (5–25% of LS) and high-LS (75–95% of LS) subsets of cells are indicated by dashed lines and are used in the following panels. (b) Density plots of normalized expression from low-LS and high-LS cells show that the constant mean is maintained by balancing the changing proportions of zeros, or near zeros in the case of scTransform, with expression shifts in normalized non-zeros. (**c**) Quantile-quantile heatmaps compare normalized expression quantiles in the high-LS (x-coordinate) and low-LS cells (y-coordinate) across genes and cell-type annotations (Supplementary Section S4.3). As in panel b, there are systematic shifts in the distributions.

The use of UMIs in the 10x Genomics and related platforms has augmented these developments in sequencing technology by tagging individual mRNA transcripts with unique cell and transcript specific identifiers. In this way, biases due to transcript length and PCR amplification have been significantly reduced (Grün *et al.*, 2014; Islam *et al.*, 2014; Tung *et al.*, 2017; Zheng *et al.*, 2017). However, technical variability in library size (LS), defined as the sum of sequenced UMIs for a given cell, remains. Consequently, normalization to remove excess variation due to LS is required to ensure accurate downstream analyses(Fig. 1). As a result, a number of normalization methods have been developed to remove the effects of LS prior to downstream analysis.

The simplest influence of LS on gene expression, the UMI counts across cells for a given gene, is the (typically) linear increase observed in expression with LS. As such, common methods for normalization are based on global scale factors which scale all reads in a cell uniformly with the goal of removing the effect of LS on average expression. In counts per ten-thousand (CPT), implemented in the popular Seurat pipeline (Butler *et al.*, 2018), each transcript within a cell is scaled such that the sum of expression across transcripts within the cell equals ten thousand; counts per million (CPM) is similar, but with the target per-cell sum equal to one million. Another widely used method, Scran (Lun *et al.*, 2016), pools counts across groups of cells to calculate scale factors which are more robust to low LS.

Bacher et al. (2017) showed that different groups of genes require different scale factors, which compromises the performance of global scale factor-based approaches. To address this, they proposed scNorm which estimates scale factors via quantile regression separately for groups of genes having similar relationships between expression and LS. While useful, their approach was developed for scRNA-seq data obtained via Fluidigm and similar protocols, and does not apply directly to UMI count data. Specifically, in addition to the distributional changes due to the deduplication of UMI data, the authors note (scNorm GitHub page and Bioconductor vignette) that for datasets with more than approximately 80% zeros, components of the model may not converge. Typical UMI datasets can have greater than 90% zeros (Townes *et al.*, 2019).

Hafemeister and Satija recently demonstrated that analysis of UMI data also requires different distributional parameters, if not different scale factors, for different groups of genes. They approach normalization as a parametric regression problem and introduce scTransform (Hafemeister and Satija, 2019) which models counts using a negative-binomial generalized linear model (glm). In scTransform, parameter estimates are smoothed across genes such that genes with similar average expression also have similar model parameters. Normalized data is then given by Pearson residuals from the regression and, as a result, the normalized expression of a typical gene has mean zero and unit variance. This approach attenuates the dependence of both the mean and variance on LS.

**Fig. 2.**
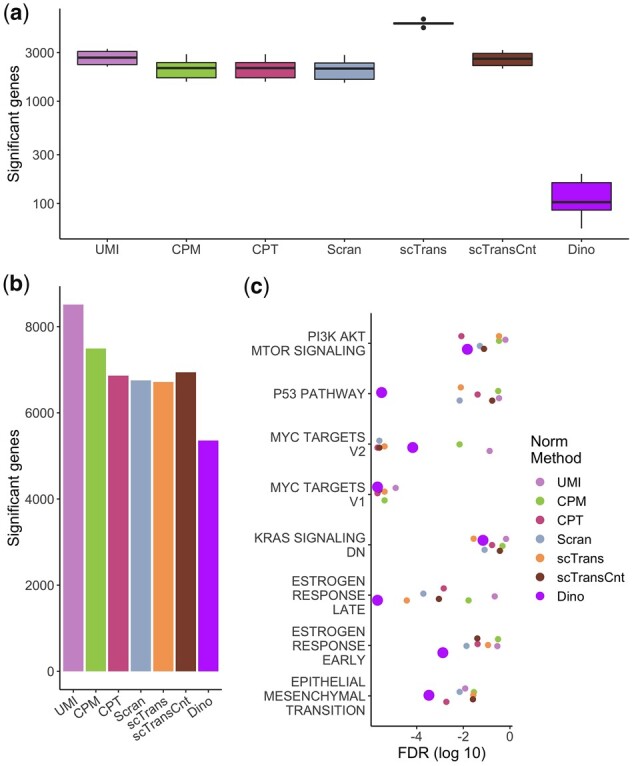
The effects of normalization on downstream DE and enrichment analysis. (**a**) Expression data from the PBMC68K_Pure dataset were normalized and genes were tested for DE using a Wilcoxon rank-sum test between low-LS and high-LS cells (5–25% and 75–95% of LS respectively) within cell-type annotations. Box plots show numbers of significant genes. Given that cells only differ in LS, significant results are considered false positives. (**b**) Expression data from the EMT dataset were analyzed using Monocle2 to identify genes with significantly variable expression over pseudo-time. Total numbers of significant genes are shown in a bar plot. (**c**) Significance values of Hallmark terms enriched for DE genes from the EMT dataset, colored for each normalization method, are plotted for the subset of terms previously identified as defining expression shifts during epithelial to mesenchymal transition.

However, variation due to LS remains in datasets normalized by these techniques. In particular, there are shifts in the distributions of normalized expression as a function of LS (Fig. 1) which then impact downstream analyses (Fig. 2). A large contributor to this observed effect is the reduction in the proportion of unnormalized zeros for a given gene as LS increases.

To address this, we present Dino, an approach that utilizes a flexible mixture of Negative Binomials model of gene expression to reconstruct full gene-specific expression distributions which are independent of LS. By giving exact zeros positive probability, the Negative Binomial components are applicable to shallow sequencing (high proportions of zeros). Additionally, the mixture component is robust to cell heterogeneity as it accommodates multiple centers of gene expression in the distribution. By directly modeling possibly heterogenous, gene-specific expression distributions, Dino outperforms competing approaches, especially for datasets in which the proportion of zeros is high, as is typical for modern, UMI-based protocols.

## 2 Materials and methods

Our proposed method for distributional normalization, Dino, reconstructs gene-specific expression distributions and provides normalized estimates of expression by constrained sampling from those distributions. Specifically, Dino assumes a hierarchical Poisson model on observed UMI counts with the distribution of Poisson means modeled as a mixture of Gammas, scaled by LS. This Gamma-Poisson model is equivalent to modeling counts as a mixture of Negative Binomials. Normalized expression is then generated by sampling from cell-specific posterior distributions of Poisson means conditioned on observed LS and UMI counts. The estimated distributions are constructed by sharing information across cells while the use of a large number of mixture components approximates the flexibility of a non-parametric approach in order to accommodate varying degrees of heterogeneity in the cell populations under study.

### 2.1 Statistical model

The count data produced by UMI sequencing protocols lend themselves naturally to a glm parameterized by LS (Anders and Huber, 2010; Hafemeister and Satija, 2019) and the random sampling of barcoded molecules from a large pool for sequencing is theoretically well modeled by independent Poisson distributions on each gene (Townes *et al.*, 2019). Furthermore, Poisson means are expected to scale proportionally with LS (Anders and Huber, 2010; Hafemeister and Satija, 2019; Lun *et al.*, 2016; Townes *et al.*, 2019), giving counts *y_gj_* from gene *g* in cell *j* the distribution *y_gj_∼f^P^(λ_gj_δ_j_)* where *f^P^* denotes a Poisson distribution with mean *λ_gj_δ_j_*. Defining *δ_j_* to be the cell-specific LS, *λ_gj_* then represents the latent level of expression for gene *g* in cell *j*, corrected for LS. Note that *λ_gj_* is cell dependent since latent levels of expression for a gene may vary across cells due to population heterogeneity and other factors. For convenience of interpretation on the *λ_gj_*, calculated LS values are scaled prior to use in the Dino model such that *δ_jMed_=1* where *jMed* is the index of the cell with the median LS across cells.

The Dino algorithm defines the distribution of *λ_gj_* across cells to be the gene-specific expression distribution of interest. If one assumes a Gamma distribution on the *λ_gj_*, then the marginal distribution of the *y_gj_* is Negative Binomial, the model assumption made by scTransform (Hafemeister and Satija, 2019), DESeq2 (Love *et al.*, 2014), ZINB-WaVE (Risso *et al.*, 2018) and SAVER (Huang *et al.*, 2018). However, we observe that unimodal distributions on the means, as implied by a single Gamma distribution on the *λ_gj_*, are often insufficient to capture the full heterogeneity present in genes of interest (Supplementary Fig. S1). Thus, to increase the accuracy with which the full gene-specific expression distribution may be estimated for all genes, Dino further assumes the *λ_gj_* arise from a mixture of Gamma distributions, and defines normalized expression as samples from the posterior distribution of the *λ_gj_*.

As distribution estimation and normalization are both performed at the gene level, the gene subscript, *g*, is hereafter dropped, and it is noted that computations are repeated across genes. This defines the Dino model as:

$$y_{j}∼f^{P}\left(\lambda_{j}\delta_{j}\right)$$

 

$$\lambda_{j}∼\sum_{K}\pi_{k}f^{G}\left(\frac{\mu_{k}}{\theta },\theta \right)$$

Here, *f^P^* is parameterized by mean *λ_j_δ_j_* and *f^G^* denotes a Gamma distribution with shape and scale parameters of *μ_k_/θ* and *θ* respectively. The mixture component probabilities, *π_k_*, are such that *Σ_K_π_k_=1*. This parameterization is chosen to define the Gamma distribution in terms of its mean, *μ_k_. K* is chosen to be sufficiently large to accommodate both cellular heterogeneity and within-cell-type over-dispersion of the *λ_j_* with respect to a single Gamma mixture component.

This approach, using a large number of components *K*, was inspired by the ash model for control of false discovery rates in statistical tests (Stephens, 2017); there, large mixtures of Normal and Uniform distributions were used to approximate arbitrary unimodal distributions with the infinite component model described as ‘a non-parametric limit’. Similar results are demonstrated by Cordy and Thomas in the context of distribution deconvolution (Cordy and Thomas, 1997). For Dino, simulated gene expression similarly demonstrates that larger estimates of *K* allow more accurate parametric estimates of the underlying distribution of UMI counts (Supplementary Figs S2 and S3). Dino sets the gene-specific value of *K* as the minimum of 100 (default) and the square root of the number of *y_j_* which are greater than zero. Testing on alternate values of *K* shows low sensitivity in downstream analysis to the choice of this parameter, assuming *K* remains sufficiently large (Supplementary Figs S4–S6). Since the negative binomial distribution can be alternately defined as the Gamma-Poisson distribution as previously discussed, this formulation has the reassuring additional interpretation of defining *y_j_* as a mixture of Negative Binomials.

### 2.2 Sampling normalized values from the posterior

The posterior distribution on *λ_j_* is straightforward to compute:

$$P\left(\lambda_{j}|y_{j},\delta_{j}\right)\propto f^{P}(\lambda_{j}\delta_{j})\sum_{k}\pi_{k}f^{G}\left(\frac{\mu_{k}}{\theta },\;\theta \right)$$

which reduces to

$$P\left(\lambda_{j}|y_{j},\delta_{j}\right)=\sum_{K}\tau_{kj}f^{G}\left(\frac{\mu_{k}}{\theta }+{\gamma y}_{j},\frac{1}{\left(\frac{1}{\theta }+{\gamma \delta }_{j}\right)}\right)$$

where *τ_kj_* is the conditional likelihood that *λ_j_* belongs to component *k* given *y_j_* and *δ_j_*, and *γ* is a concentration parameter. Our testing (Supplementary Figs S7–S9) has demonstrated that resampling from the strict posterior distribution can lead to excessive variance in the normalized expression which can obscure biological features of interest. Therefore, *γ* is included to both reduce the normalized variance and center normalized values around their corresponding scale-factor values.

Default values of *γ = 15* have proven successful. This adjustment can be seen as a bias in the normalized values toward a scale-factor version of normalization, since, in the limit of *γ*, the normalized expression for cell *j* converges to y*_j_/δ_j_*. A modified expectation-maximation (EM) algorithm (Jamshidian and Jennrich, 1997) is used to estimate *μ_k_* and *τ_kj_* (Supplementary Section S1.1). Separate values of *θ* are estimated for each gene based on gamma kernel density estimation (Chen, 2000) (Supplementary Section S1.2). To accommodate slight deviation from strict expression scaling with LS, adjustments to *δ_j_* are made prior to model parameter estimation (Supplementary Section S1.3). Finally, parameter initialization relies on a modified application of quantile regression (Branham, 1982; Powell, 1984, 1986) (Supplementary Section S1.4). An alternate (non-default) sampling method designed to preserve expression ranks is also available to the user (Supplementary Section S1.5).

### 2.3 Datasets

Results from six publicly available datasets are evaluated: PBMC68K_Pure, PBMC5K_Prot, MaltTumor10K, MouseBrain, PBMC68K and EMT. Where applicable, analyzed expression was derived from unfiltered gene-barcode matrices, with empty droplets removed by the tools in the R package DropletUtils (Lun et al., 2019).

PBMC68K_Pure is a partner dataset to PBMC68K (Zheng *et al.*, 2017) produced by purifying peripheral blood mononuclear cells (PBMCs) into 10 cell types through the use of cell-type specific isolation kits and separately sequencing each group. These cell-type annotations are considered here as ground truth when evaluating the effects of normalization on downstream clustering. For increased accuracy, the six cell-types for which tSNE plots do not separate into sub-groups (Van Der Maaten, 2014; van der Maaten and Hinton, 2008) were subset, denoting low-heterogeneity references: CD4+ T Helper, CD4+/CD25 T Reg, CD4+/CD45RA+/CD25- Naive T, CD4+/CD45RO+ Memory, CD56+ NK and CD8+/CD45RA+ Naive Cytotoxic. Zheng *et al.* identify these particular six cell-types as demonstrating little sub-structure (Zheng *et al.*, 2017).

EMT is a dataset of 5004 MCF10A mammary epithelial cells induced to undergo spontaneous epithelial to mesenchymal transitions through the cellular detection of neighboring unoccupied space (McFaline-Figueroa *et al.*, 2019). This spatial effect allowed the authors to dissect an inner region *a priori* expected to be primarily epithelial cells and an outer region *a priori* expected to be primarily mesenchymal cells. Cells from each region were then sequenced separately. From all the data published by the authors, the EMT dataset we consider is denoted ‘Mock’ in the barcode metadata. Included in the initial publication, the authors describe eight gene sets from the Hallmark collection (Liberzon *et al.*, 2015) which they consider to be significantly enriched for activity during the epithelial to mesenchymal transition. We take this set of terms as a ground truth for assessing the power of analysis based on each normalization method.

Results from the PBMC68K_Pure and EMT datasets are shown in the manuscript while results from other datasets are provided in the supplement. Details on each of the datasets as well as their pre-processing are provided in Supplementary Section S2.

The case study datasets are also used to generate simulated datasets with expression profiles designed to closely mirror individual experimentally observed cells. In brief, for a given case study dataset, unsupervised clustering is used to define clusters. Two cells within the same cluster and with similar LS are sampled and UMI counts are summed across the two cells to make a pseudo-cell. Expression from each gene of this pseudo-cell is then down-sampled using a binomial distribution to generate two new simulated cells which differ in LS due to different binomial probability parameters. Constant binomial probabilities across genes results in equivalent expressed (EE) between the two simulated cells, after accounting for LS, for all but 10 genes for which differential expression (DE) is induced by alteration of the binomial probability. Here EE is used to denote a gene whose average expression is equal across two cells (or two groups of cells) which have been properly normalized for LS. DE denotes a difference in means between two groups unless otherwise specified. This process of constructing and down-sampling pseudo-cells is repeated to generate a collection of cells with the same set of EE and DE genes, but varying in LS; the process is repeated again for other clusters to simulate subpopulation heterogeneity. These simulated datasets are then used to quantify power and false positive rates for DE testing following different normalization methods. Full simulation details are described in Supplementary Section S3. Different methods of pre-clustering cells yield similar results (Supplementary Figs S10–S12).

### 2.4 Application of normalization methods

For each dataset considered, normalized estimates of expression were obtained from Dino (v0.6.2), Scran (v1.16.0), scTransform (v0.2.1), CPM and CPT. We also consider un-normalized UMIs for reference. Further information on package defaults, annotation versions and other software is given in Supplementary Section S4. The implementation of scTransform provides both normalized expression in terms of regression residuals (recommended for most analysis applications by the authors) and normalized expression in terms of a corrected UMI counts matrix. We consider both in this manuscript and refer to the residuals matrix as scTrans and the corrected counts matrix as scTransCnt.

Given its additional complexity, Dino is generally more computationally demanding than existing methods. However, it remains competitive even in the context of the large datasets currently being generated. We performed a test of computational intensity on a 2017 MacBook Pro with a 3.1 GHz quad-core CPU and 16 GB of RAM. Optimizations in the Dino package, including the estimation of model parameters on a default subset of 10 000 cells when input data exceeds that number, allow Dino to successfully normalized datasets which are too large for other applications to handle on this hardware (Supplementary Fig. S13).

## 3 Results

### 3.1 LS-dependent patterns in normalized data

To compare Dino, Scran and scTransform, we normalized the PBMC68K_Pure data using each method. Given the cell-type purification of this dataset, cells within a given annotation are expected to be largely homogeneous and, consequently, should exhibit little difference in expression among cells following normalization. Examination of the normalized expression between low-LS and high-LS CD4+/CD45RO+ memory cells shows that existing methods exhibit significant LS-dependent effects. As shown in Figure 1a, for a normalized gene to maintain average expression that is relatively independent of LS, the higher proportion of zeros in the low-LS group is balanced by inflation of the non-zeros. This leads to shifts in the densities of the normalized non-zeros between low and high-LS cells, as shown in Figure 1b. To evaluate this effect across all genes, we compare the quantiles in low-LS and high-LS cells for a random sample of genes across all cell-types in PBMC68K_Pure through a modified Q-Q plot. As with traditional Q-Q plots, high frequency off the diagonal in Figure 1c indicates differences in the distribution of normalized expression between low-LS and high-LS cells (Supplementary Section S4.3). Scran and scTransform demonstrate systematic shifts in the distribution of normalized expression as a function of LS. Normalized expression from Dino, however, mitigates these effects, producing more equivalent expression distributions across low and high-LS cells. We observe similar results for other case study datasets (Supplementary Fig. S14).

### 3.2 Effects of normalization on differential expression analysis

To evaluate the extent to which LS-dependent differences in the expression distributions affect downstream differential expression (DE) analysis, we used the Wilcoxon rank-sum test, the default test in the Seurat pipeline as of writing, to identify significantly DE genes between low and high-LS cells. Tests were conducted within each of the six annotated cell-types in the PBMC68K_Pure dataset, resulting in six separate measures of significant genes. Figure 2a demonstrates that many genes are identified as significant by most methods. As each cell-type is homogeneous by construction, differences in LS are not expected to indicate true biological variability and significant results are thus considered false positives. These results are consistent with the changes in normalized expression distributions observed in Figure 1 as the Wilcoxon test, as well as others widely used in scRNA-seq analysis such as MAST (Finak *et al.*, 2015), is susceptible to differences in distribution, not merely differences in means. Similar results are observed for the MaltTumor10K and PBMC5K_Prot datasets compared against pseudo-annotations of cell-type (Supplementary Fig. S15). A control comparison further indicates that observed high rates of significant genes are largely due to false positives. When comparisons are made between randomly selected cells as opposed to low-LS versus high-LS cells, exactly 0 genes are significant (*P*-adj ≤ 1e-2) following a test of DE.

To evaluate DE analysis in the positive case where DE genes are expected to exist, we considered a case study dataset of cells undergoing spontaneous epithelial to mesenchymal transitions from the EMT dataset (McFaline-Figueroa *et al.*, 2019). Following the originally published analysis pipeline, we used Monocle2 (Qiu *et al.*, 2017; Trapnell *et al.*, 2014) to construct a pseudo-time model of the experimentally induced differentiation tree (Supplementary Section S4.4). Cells along the primary branch of the differentiation tree were tested for DE, here defined as having a significant change in expression over pseudo-time.

As with the negative control examples of Figure 2a and Supplementary Figure S15, Dino normalized data results in the fewest significant genes (Fig. 2b). Results from an enrichment analysis (Fig. 2c) suggest that the reduction in the number of significant genes found by Dino is likely due to a reduction in false positives, as with the negative control, rather than an undesirable reduction in power. Specifically, we performed gene set enrichment analysis (GSEA) on the Hallmark collection of gene sets (Liberzon *et al.*, 2015). Enrichment significance values are plotted in Figure 2c for the eight terms identified by McFaline-Figueroa *et al.* as significant markers of EMT activity. Dino normalization results in competitive significance in four of the eight terms and shows the highest GSEA significance in the remaining four terms. Notably, Dino shows improved enrichment results for the term defining the epithelial to mesenchymal transition; the Dino adjusted *P*-value (*P*-adj = 3.3e-4) is close to an order of magnitude more significant than the nearest alternate method (CPT, *P*-adj = 1.9e-3).

**Fig. 3.**
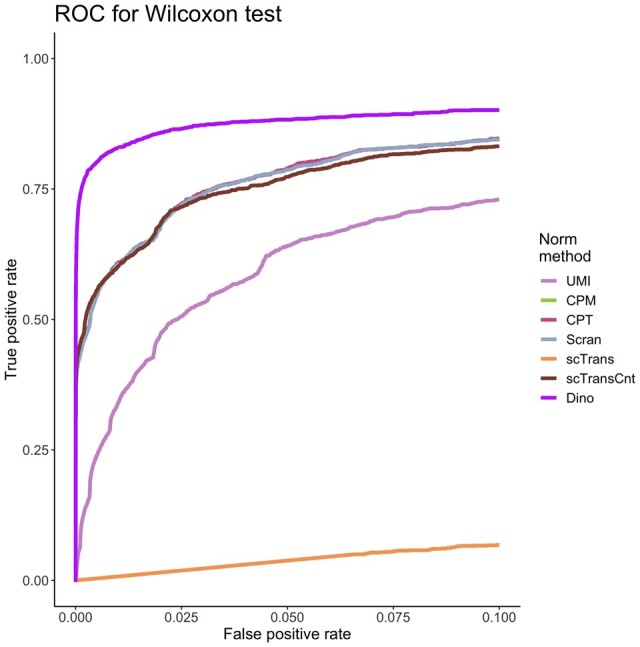
The effects of normalization on downstream DE analysis. Simulated data based on the PBMC68K_Pure dataset were normalized using each method. ROC curves colored by normalization method define the relationship between average TPR and average FPR for a Wilcoxon rank-sum test, where the average is calculated across 30 simulated datasets.

Simulated datasets provide further insights consistent with the case study results regarding DE analysis. To quantify the relationship between true positive rates (TPR) and false positive rates (FPR), we simulated heterogenous cell populations from experimentally observed expression with known DE and EE genes (Supplementary Section S3). Following normalization, DE analysis was conducted using a Wilcoxon rank-sum test. Figure 3 plots average ROC curves for each normalization method where the average is taken over 30 datasets simulated from the PBMC68K_Pure dataset. For most methods, it can be observed that high power is confounded by high FPRs. As with the negative control, however, Dino controls FPR to much lower levels. Repeated simulations and analysis across the other five datasets considered yield similar results, as does analysis based on MAST (Supplementary Figs S16 and S17). Performance is more similar across methods when DE tests are conducted via *t*-tests, since *t*-tests identify shifts in means and are not sensitive to other differences in distributions (Supplementary Fig. S18). Point estimation of TPR/FPR controlling adjusted *P*-values at 0.01 through the method of Benjamini and Hochberg (Benjamini and Hochberg, 1995) supports the ROC plots (Supplementary Tables S1–S3).

### 3.3 Effects of normalization on clustering

**Fig. 4.**
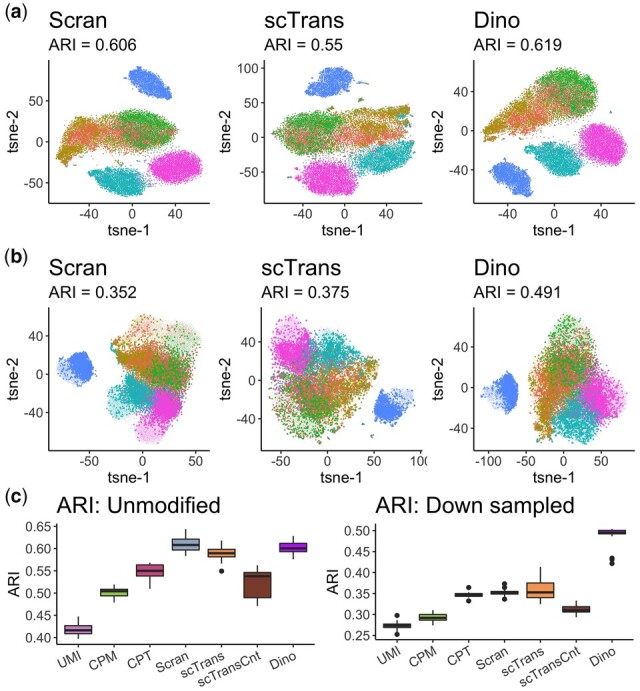
The effects of normalization on clustering. (**a**) tSNE plots of normalized PBMC68K_Pure data, colored by 6 cell-type annotations, show similarly high clustering accuracy across methods. (**b**) The same clustering plots as in (a), but with half the data down-sampled (down-sampled cells in bold) prior to normalization to produce greater differences in LS. (**c**) Boxplots of ARIs for multiple un-modified and down-sampled datasets across 24 samples of 25 thousand cells from the PBMC68K_Pure dataset.

The effect of normalization on clustering was evaluated by comparing clusters derived from data normalized by each method with the annotations in the PBMC68K_Pure dataset using the adjusted Rand index (ARI). As shown in Figure 4, for one sub-sample of cells, Dino, Scran and scTrans outperform other methods; Dino shows slightly although not significantly improved performance over scTrans and Scran (Fig. 4a). To exacerbate the differences in sequenced LS and thereby highlight the effect of differences in LS on clustering, we randomly down-sampled half of the 25000 cells to 25% of their original LS. Figure 4b shows the derived clusters with the down-sampled cells (bold) compared to the unmodified cells. Some effect of LS is observed in the clustering, and ARIs for all normalization methods decreased, as expected. However, Dino normalized data retains a more accurate differentiation between cells with an ARI of 0.491 compared to 0.375 and 0.352 for scTrans and Scran, respectively.

Figure 4c shows results from the analysis repeated across 24 samples of 25 000 cells. As with the sample shown in Figure 4a, Dino and Scran perform comparably (medians of 0.601 and 0.608 respectively), and uniformly better than scTrans (median of 0.590). In the down-sampled case, Dino performs significantly better than competing methods (*P* < 2.2e-16 under a *t*-test). Similar results are observed for the MaltTumor10K and PBMC5k_Prot datasets (Supplementary Figs S19 and S20).

## 4 Discussion

Droplet-based, UMI protocols provide unprecedented cellular resolution in expression profiling. However, the use of UMIs does not remove the need for effective normalization in the analysis of such data, and the extreme sparsity of these high-throughput experiments introduces new challenges for LS correction. Dino adapts to these challenges by correcting the full expression distribution of each gene for LS-dependent variation, rather than only correcting mean expression as with most existing methods. This approach increases both power and precision in down-stream analysis.

As noted in methods, our approach assumes that latent counts for a given gene in a given cell are Poisson distributed, with latent means described by a Gamma mixture. The Gamma-Poisson framework is common in single-cell RNA-seq approaches (Hafemeister and Satija, 2019; Huang *et al.*, 2018; Love *et al.*, 2014; Risso *et al.*, 2018); however, the Gamma mixture component introduced here directly accommodates heterogeneity which leads to improved operating characteristics in a number of settings. Dino normalizes observations by resampling from the gene-specific posterior distribution to provide normalized estimates of expression. While the default approach presented here provides unconstrained sampling up to differences in sample-specific posteriors as detailed in methods, a user may choose to perform an alternate restricted quantile sampling that aims to preserve un-normalized expression ordering in the normalized data (Supplementary Section S1.5). Other measures from the posterior could potentially be obtained (e.g. the posterior mean or median). However, this is not recommended in practice—and not an available option in the distributed package—as doing so preserves the LS-dependent variation in distribution observed in existing methods, especially for low and moderately expressed genes (Supplementary Fig. S21).

In some cases, such as when a cell type under study down/up-regulates a significant majority of DE genes compared to other cell types being sequenced, a researcher may find that LS is correlated with true biological variability. In these situations, normalization to remove the effects of LS would further remove biological variation of interest. To accommodate these situations, Dino allows the user to supply alternate, cell-specific size factors, such as those estimated by Scran, as the measure of technical nuisance variation. Implementation details are available in the package documentation and vignette.

In contrast to existing methods, the resampling performed by Dino accommodates the varying proportions of zeros across LS, reducing or removing the effect of LS on the full expression distribution. In addition, by resampling from the full gene-specific distribution, Dino produces greater homogeneity of normalized expression across LS within cell type, and therefore leads to more accurate downstream analyses that are robust to heterogeneous cell populations.

## Supplementary Material

btab450_Supplementary_Data
