# Supplementary material for: Normalization by distributional resampling of high throughput single-cell RNA-sequencing data
Source: Bioinformatics. 2021 Jun 19;37(22):4123–8. doi: 10.1093/bioinformatics/btab450 (PMC9502161; doi:10.1093/bioinformatics/btab450)
Supplement: btab450_Supplementary_Data [file btab450_supplementary_data.docx]

Supplement to *Normalization by distributional resampling of high throughput single-cell RNA-sequencing data* by Jared Brown, Zijian Ni, Chitrasen Mohanty, Rhonda Bacher, and Christina Kendziorski

# S1 Dino algorithm – additional details

## S1.1 EM iterations

To speed computation, the distribution on the *λ_j_* is approximated by a mixture of point masses: *λj~Σ_k_π_k_f^δ^(μ_k_)* where *f^δ^(x)* is a delta function centered at *x*. The model then reduces to a mixture of Poissons, and so the expectation maximization (EM) algorithm has fast, closed form iterations for estimating *π_k_* and *μ_k_*, which are further accelerated using a quasi-Newton adjustment to the EM update (Jamshidian and Jennrich, 1997). In brief, this quasi-Newton adjustment replaces the usual EM update, *g(τ)*, where *τ=[π, μ]*, with a “corrected” EM step: *g(τ)-Sgl(τ)* where *S* is an estimate of the inverse Hessian of the likelihood function based on the ﻿Broyden-Fletcher- Goldfarb-Shanno symmetric rank 2 update and *gl(τ)* is the gradient of the likelihood function. In our application, the default step length of 1, applied to the entire corrected EM step, is adjusted as necessary to accord with the strong Wolfe conditions.

The EM algorithm requires initialization of the means *μ_k_* and the cluster occupancy rates *π_k_*. For simplicity, the rates *π_k_* are initialized as *1/K*, and the *μ_k_* are initialized both to remove unnecessary iterations in the model fitting and to conform with these initial rates. To accomplish this, we derive the starting *μ_k_* values from equal spacings on an estimate of the cumulative distribution function (CDF) of the *y_j_* at unit library size (LS), *δ=1*. Details of estimating the CDF are provided below in Section S1.4.

Following convergence, the estimates of *μ_k_* are used to parameterize the Dino mixture of Gammas. In the posterior distribution used for resampling of normalized expression values, the conditional likelihood of component membership given by *τ_kj_* is given by the conditional likelihood that observation *y_j_* was sampled from Poisson component *k* from the above mixture of point masses used in the EM algorithm. This value, *τ_kj_*, is directly estimated as a step in the EM algorithm.

## S1.2 Dispersion parameter estimation

Following the fitting of the EM model in S1.1, Gamma kernel density estimation is used to estimate the remaining Gamma parameter, *θ*, given the estimates of *μ_k_*. In particular, given an observed sample *{μ_k_}*, the underlying distribution of the *{μ_k_}* can be estimated as a mixture of Gammas parameterized by shape *μ_k_/θ* and scale *θ* (Chen, 2000). In our application, *θ* is estimated using a kernel density bandwidth estimator (R function bw.ucv by default) applied to the estimates of *μ_k_*. The resulting bandwidth estimate of *θ* is then trimmed to a maximum of 1 such that the variance of the estimated Gamma components not exceed the variance of the Poisson components used in the original EM estimation of the means *μ_k_*.

## S1.3 LS adjustment

LS for cell *j*, *δ_j_*, is typically estimated as the sum of UMIs across genes for a given cell, or more generally, as the sum of aligned reads for a given sample. Given this, a cell with zero LS should have zero counts, and a glm fitted to expression vs. LS should have a zero intercept and linear relationship with LS, which corresponds to a slope coefficient of 1 under the usual log link function. Such values, however, are not universally observed, although calculated coefficients are generally close to 1 (Bacher *et al.*, 2017; Hafemeister and Satija, 2019). Note that, as mentioned in the main text, in our application LS values are scaled such that the median LS is 1, i.e., *δ_jMed_=1*.

To obtain improved estimates of *δ_j_*, we estimate the slope (under the log link-function) between counts and LS. Specifically, we randomly sample a subset of genes (10,000 by default), with sampling weighted by the inverse density of the gene expression level to ensure proper representation of the (relatively rare) high expressing genes. To accommodate sub-population heterogeneity, we perform clustering on these data using the methods implemented in scran (Lun *et al.*, 2016), and fit a Poisson glm to each gene, generating a unique slope estimate for each gene with each cluster of cells getting a unique intercept. A dataset-wide slope, *s*, is computed as the mean of fitted slopes to each gene; *s* is close to 1 for most datasets. Log-LS estimates, *δ_j_’*, are then corrected prior to use in the above mixture model as *δ_j_’=sδ_j_’-(s-1)*. To avoid the rare occurrence of negative LSs post-transformation, *s* is bounded above and below such that the minimum transformed LS is at least *exp(-3)* times its original value.

## S1.4 Estimation of the gene-specific CDF by cLAD regression

Section S1.1 mentions that the initial values of the means, *μ_k_*_,_ are taken from equal spacings along an estimate of the CDF of the counts *y_j_* at LS *δ=1*. Unfortunately, estimation of this CDF is non-trivial; standard formulas for the empirical CDF (eCDF) are confounded by the need to remove the effect of LS prior to computation (normalization) and, more importantly, are further confounded by the truncation of *y_j_* at 0.

To solve this problem, we demonstrate that this eCDF can, in fact, be efficiently calculated for each gene by a modified application of censored least absolute deviations (cLAD) regression (Powell, 1984, 1986) at a carefully chosen grid of given intercepts. cLAD regression has the benefit here of solving for linear functions of quantiles in data – hence its alternate name, quantile regression. The pairing of fitted intercepts (quantiles) and corresponding percentiles then define points along the desired eCDF.

Regression calculations are conducted on the log-log scale, so the both the LS values, *δ_i_*, and the counts, *y_j_*, are log transformed with a floor of *log(0.999)* for the observed counts; *d_j_=log(δ_j_)*, *z_j_=max{log(y_j_), log(0.999)}*. The implicit addition of a *≈1* pseudo count to the observed zeros prior to log transformation is motivated by the fact that computations are performed using censored quantile regression, which can be written as standard quantile (LAD) regression on the subset of data to the right of the intersection of the regression curve and censoring threshold (Powell, 1986). In particular, the regression model for the observed data is:

$$z_{j}=\max\left\{ \log\left( 0.999 \right),\beta_{0}+d_{j}+\epsilon_{j} \right\}$$

where *β_0_* is some gene-specific intercept and *ϵ_j_* is a random error term following some, possibly complex distribution. For example, in the presence of population heterogeneity, the *ϵ_j_* might follow a bi-modal distribution. Placing the zeros at *log(0.999)* allows the regression solutions to be defined in terms of the strictly positive data as the censoring threshold is placed just below log(1). The choice of a constant slope term (the implicit coefficient of 1 on the LS term), while mathematically convenient in the following, is also not unreasonable. On the multiplicative (log) scale, expression should have about a slope one relationship with LS across all genes, recalling the above comment that both counts and LS are log-transformed for the cLAD regression.

**Estimation of expression distribution quantiles:** The gene-specific eCDFs to be computed are denoted by vectors of quantiles and the associated estimated percentiles. To calculate the sample quantiles and percentiles of the eCDF, and as a natural extension of the censored linear model of the data distribution, the mathematics of censored least absolute deviations regression (cLAD) are adopted. The cLAD minimization problem is

$$\min_{\beta_{0}} \left\{ \sum_{j} p_{\pi}\left[ z_{j}-\left( \beta_{0}+z_{j} \right)^{*} \right] \right\}$$

where *p_π_(x)=(π-𝕀(x<0))(x)*, *(x)^*^=max{log(0.999), x)*, and *π* is some percentile (eg. *π=0.5* for median quantile regression). It has previously been shown that cLAD regression has beneficial properties, particularly consistency in the presence of only weakly defined residual distributions (Powell, 1984, 1986). Additionally, study of the solution set to cLAD regression has shown that solution coefficients define lines which pass through at least as many data points as there are free parameters (Branham, R. L., 1982). Given this, the regression problem can be significantly reduced. Given a fixed percentile, *π*, the set of possible solutions is confined to a set of parallel lines – one intersecting each point in the data set – which is finite for finite data. The regression problem is then to determine which line, uniquely defined by its intercept *β_0_*, minimizes the loss function.

As such, the set of quantiles defining the eCDF is easily defined. In particular, the eCDF quantiles are the set of intercepts, *β_0j_*, defining the lines passing through each of the data points. The intercepts/quantiles refer to the previously mentioned “grid of given intercepts.” Since this set of intercepts contains the minimizing solution regardless of the choice of *π* in the regression problem, any additional quantiles would by definition be redundant. Specifically, the set of quantiles *{q_j_}* is defined as:

$$\left\{ q_{j} \right\}=\left\{ \beta_{0j} \right\}=\{z_{j}-d_{j}\}$$

**Estimation of expression distribution percentiles:** For simplicity, consider a highly expressed gene for which there are no observed zero counts prior to log transformation and suppose without loss of generality that the quantiles *{q_i_}* are all unique and that the indices *j* are of decreasing order such that *q_j_>q_j+1_∀j*, *q_1_=max{q_j_}*. In this case, computing the percentiles associated with each quantile is trivial and follows the standard eCDF formula:

$$p_{j}=\frac{1}{n}\sum_{i} \mathbb{I}\left( q_{i}\leq q_{j} \right)=\frac{n-j+1}{n}$$

where *n=|{q_j_}|*. The more general case where a gene may contain zeros, possibly many zeros, is more complicated. However, given the assumption that the residual distribution (*ϵ_j_*) is constant in LS, a simple and analogous solution exists:

$$p_{j}=\frac{n_{j}-\left| \left\{ q_{i} \right\}_{j} \right|}{n_{j}}$$

where

$$\Lambda_{j}=\left\{ i:\delta_{i}\geq\delta_{j}-\left( z_{j}-\log\left( 0.999 \right) \right) \right\}$$

$$n_{j}=\left| \Lambda_{j} \right|$$

$$\left\{ q_{i} \right\}_{j}=\left\{ q_{i}:q_{i}>q_{j}, i\in\Lambda_{j} \right\}$$

The linear interpolation of the set $\left\{ \left( q_{j},p_{j} \right) \right\}$defines an eCDF from which the *π_j_* can be initialized.

**Monotonicity correction:** In practice, this estimate of the percentiles *p_j_* can become unstable for high *j* (when *n_j_* becomes small). Additionally, some forms of population heterogeneity can cause estimation problems, especially as they can violate the assumption of constant residual distribution. Here we derive the above formulation for the percentiles as well as corrections for these situations.

Recall the example of the highly expressing gene mentioned above. As noted, it is trivial to estimate *p_j_*, and guaranteed that the *p_j_* will be both unique and monotone. To facilitate the discussion of the more general case (where there are zero counts), consider this ideal problem (no zeros) in the context of standard LAD regression. For notational convenience, make the following definitions:

$$SUD_{j}^{'}\left( \beta_{0j} \right):=\sum_{\{i:z_{i}>\left( \beta_{0j}+d_{i} \right)\}} \left[ z_{i}-\left( \beta_{0j}+d_{i} \right) \right]$$

$$SLD_{j}^{'}\left( \beta_{0j} \right):=\sum_{\{i:z_{i}\leq\left( \beta_{0j}+d_{i} \right)\}} \left[ \left( \beta_{0j}+d_{i} \right)-z_{i} \right]$$

where the abbreviations denote “sum of upper deviations” and “sum of lower deviations” respectively across the collection of cells indexed by *i*.

To solve for a percentile given a quantile, the previous regression problem is inverted – and expanded to remove the *p_π_(⋅ )* notation – to find the set

$$\left\{ \pi:\beta_{0j}=\min_{\beta_{0}} \left( \pi SUD_{j}^{'}\left( \beta_{0} \right)+\left( 1-\pi\right)SLD_{j}^{'}\left( \beta_{0} \right) \right) \right\}$$

Note that *SUD_1_^’^=0* and *SLD_n_^’^=0* and that *SUD_j_^’^* (*SLD_j_^’^*) are increasing (decreasing) in *j*. Additionally, a linear interpolation of *SUD_j_^’^* (*SLD_j_^’^*) would have positive (negative) derivatives. Thus, the surface of the convex hull of the set *{(SUD_j_^’^, SLD_j_^’^)}* contains all points within the set. This means that for each *β_0j_*, there exists some unique *π_j_* for which *β_0j_* is the minimizer of the usual, non-inverted, LAD problem. Specifically, a minimizing *π_j_* is one such that the line *π_j_SUD_j_^’^-(1-π_j_)SLD_j_^’^=c*, for some constant *c*, is a sub-tangent of the linear interpolation of *{(SUD_j_^’^, SLD_j_^’^)}* at the relevant point.

This can be demonstrated as follows: suppose *-π_j_/(1-π_j_)* is a slope in the sub-derivative of the linear interpolation at point *(SUD_j_^’^, SLD_j_^’^)* so that the convex combination of sums of deviations takes some value, *π_j_SUD_j_^’^+(1-π_j_)SLD_j_^’^=b*. Consider then the point *(SUD_j+1_^’^, SLD_j+1_^’^)* and define *(dU,dL)≔(SUD_j+1_^’^,SLD_j+1_^’^)-(SUD_j_^’^,SLD_j_^’^)* where *dU,-dL>0* so the convex combination at *j+1* can be written as *π­_j_(SUD_j_^’^+dU)+(1-π_j_)(SLD_j_^’^+dL)=b+π_j_dU+(1-π_j_)dL*. Since *-π­_j_/(1-π_j_)* is in the sub-derivative, it is the case that

$$-\frac{\pi_{j}}{\left( 1-\pi_{j} \right)}\leq\frac{dL}{dU}\Longrightarrow\pi_{j}dU+\left( 1-\pi_{j} \right)dL\geq0$$

with equality only if *-π_j_/(1-π­_j_)* is the maximal sub-derivative, showing *β_0(j+1)_* is not a solution for the LAD problem given weight *π_j_* excepting only the minimal *π_j_* allowed by the sub-derivative. A similar result holds for point *j-1*.

Therefore, to solve for a percentile *p_j_*, one can consider the derivatives of the sums of deviations parameterized by the intercept *β_0j_*

$$dSUD_{j}^{'}=\frac{d}{d\left( \beta_{0j} \right)}SUD_{j}^{'}=-\left| \{j:z_{j}>\left( \beta_{0j}+d_{j} \right)\} \right| =-\left( j-1 \right)$$

$$dSLD_{j}^{'}=\frac{d}{d\left( \beta_{0j} \right)}SLD_{j}^{'}=\left| \{j:z_{j}\leq\left( \beta_{0j}+d_{j} \right)\} \right|=\left( n-j+1 \right)$$

where the second equalities follow from the uniqueness and ordering of the indices *j*.

Then the slope of one possible subtangent in terms of *π_j_* is

$$-\frac{\pi_{j}}{\left( 1-\pi_{j} \right)}=\frac{dSLD_{j}^{'}}{dSUD_{j}^{'}}=-\frac{\left( n-j+1 \right)}{\left( j-1 \right)}$$

so

$$p_{j}=\pi_{j}=\frac{-\frac{dSLD_{j}^{'}}{dSUD_{j}^{'}}}{1-\frac{dSLD_{j}^{'}}{dSUD_{j}^{'}}}=\frac{\left( n-j+1 \right)}{\left( j-1 \right)+\left( n-j+1 \right)}=\frac{n-j+1}{n}$$

which is the same result as at the beginning of this section.

To generalize solving for percentiles *{p_j_}* in the context of cLAD regression, one need only make a few modifications to the above results. First, define

$$SUD_{j}\left( \beta_{0j} \right)=\sum_{z_{i}\geq\left( \beta_{0j}+d_{i} \right)^{*}} \left[ z_{i}-\left( \beta_{0j}+d_{i} \right)^{*} \right]$$

$$SLD_{j}\left( \beta_{0j} \right)=\sum_{z_{i}<\left( \beta_{0j}+d_{i} \right)^{*}} \left[ \left( \beta_{0j}+d_{i} \right)^{*}-z_{i} \right]$$

for the censoring function *(x)^*^*. Then the percentile problem seeks to find sets of a familiar form:

$$\left\{ \pi:\beta_{0j}=\min_{\beta_{0}} \left( \pi SUD_{j}\left( \beta_{0} \right)+\left( 1-\pi\right)SLD_{j}\left( \beta_{0} \right) \right) \right\}$$

with a familiar solution,

$$-\frac{\pi_{j}}{\left( 1-\pi_{j} \right)}=\frac{dSLD_{j}}{dSUD_{j}}$$

assuming the same convexity conditions hold for the set *{(SUD_j_,SLD_j_)}.*

In the presence of censoring, the convexity conditions may not hold. A correction to enforce convexity is discussed later in the section. The main difference between the general result which accommodates censoring and that for LAD regression is in the precise formulation of the derivatives of the sums of deviations.

$$dSUD_{j}=\frac{d}{d\left( \beta_{0j} \right)}SUD_{j}$$

$$=-\left| \left\{ j:z_{j}>\left( \beta_{0j}+d_{j} \right)^{*},d_{j}\geq\beta_{0j}-\log\left( 0.999 \right) \right\} \right|$$

$$dSLD_{j}=\frac{d}{d\left( \beta_{0j} \right)}SLD_{j}$$

$$=\left| \left\{ j:z_{j}\leq\left( \beta_{0j}+d_{j} \right)^{*},d_{j}\geq\beta_{0j}-\log\left( 0.999 \right) \right\} \right|$$

These derivatives do have a similar interpretation to those of the previous section, however. Specifically, up to a sign change, they are the number of observations above/below the regression line under consideration which *also* have LS above the point where that regression line hits the censoring threshold of *log(0.999)*.

This gives a convenient interpretation to the solution for *p_j_* as well. The solution itself is

$$p_{j}=\frac{dSLD_{j}}{dSLD_{j}-dSUD_{j}}$$

which is simply the empirical percentile from before, but computed on the subset of observations with LS above the point where the regression line becomes censored. This is consistent with the result from Powell that cLAD regression is equivalent to LAD regression performed on the subset of data for which the probability of censoring is uniformly no greater than the regression percentile *π* and at some covariates the probability of censoring is strictly less than *π*.

It was previously noted that the censored data do not guarantee the convexity conditions on the set of upper and lower deviations as is the case in traditional LAD regression. This can occur stochastically in the lower quantiles when there are few data points from which to estimate the percentiles. This can also occur systematically when the observed expression is correlated with LS as may occur when sub-populations of cells express in aggregate at different levels.

To correct for both of these issues simultaneously, a monotonicity condition is imposed on the estimated *p_j_*. First, the *p_j_* are computed only on the subset of upper/lower deviations that exist on the edge of the convex hull of *{SUD_j_, SLD_j_}*. Following computation of percentiles on this subset of quantiles, percentiles are adjusted such that differences between adjacent percentiles are bounded above and below. The bounds are as follows:

$$p_{j}-p_{j+1}\geq\frac{\sum_{k} z_{k}>\left( \beta_{0j}+d_{k} \right)^{*}-\sum_{k} z_{k}>\left( \beta_{0j+1}+d_{k} \right)^{*}}{n}$$

$$p_{j}-p_{j+1}\leq p_{j}-\frac{\sum_{k} z_{k}<\left( \beta_{0j+1}+d_{k} \right)^{*}}{n}$$

## S1.5 Restricted Quantile Sampling (RQS)

As an alternate to the default normalization by resampling from gene/cell-specific posterior distributions, Dino also supports a method of normalization designed to maximally preserve the order of observed un-normalized expression in the normalized matrix. The method, which we call restricted quantile sampling (RQS), is based on the idea that, if two cells have the same library size, then for a given gene, the cell with strictly greater observed expression in the un-normalized data should also have strictly greater expression following normalization.

This alternate approach (and its name) derives from the observation that, when sampling from a discrete distribution – such has the mixture of Poissons estimated by the Dino EM algorithm (S1.1 Section) – by the method of inverse transform sampling, a band of sampled percentiles all result in the same sampled quantile. This band then defines lower and upper bounds on the percentiles which could have resulted in the observed, un-normalized expression counts. Letting *F(q|δ_j_)* denote the estimated mixture of Poissons cumulative mass function for a given LS, *δ_j_*, these bounds are then estimated as:

$$L_{j}:=\left\{ \begin{matrix} 0 & if y_{gj}=0 \\ F\left( y_{gj}-1 | \delta_{j} \right) & else \end{matrix} \right.$$

$$H_{j}:=F(y_{gj}|\delta_{j})$$

Letting *G^-1^(p)* denote the estimated mixture of Gammas inverse cumulative distribution function at the target LS and letting *U(l,h)* denote the uniform density function between lower bound *L* and upper bound *H*, the normalized expression by the RQS method is defined as:

$$\hat{y}_{j}:=G^{-1}(p_{j})$$

$$p_{j}\sim U(L_{j},H_{j})$$

# S2 Datasets

## PBMC_Pure

*PBMC68K_Pure* is a partner dataset to PBMC68K (Zheng et al., 2017) produced by purifying peripheral blood mononuclear cells (PBMCs) into 10 cell types through the use of cell-type specific isolation kits and separately sequencing each group. One group was then computationally separated into two resulting in 11 annotated cell-types. These cell-type annotations are considered here as ground truth when evaluating the effects of normalization on downstream clustering. For increased accuracy, the six cell-types for which tSNE plots do not separate into sub-groups (van der Maaten and Hinton, 2008; Van Der Maaten, 2014) were subset: CD4+ T Helper2, CD4+/CD25 T Reg, CD4+/CD45RA+/CD25- Naive T, CD4+/CD45RO+ Memory, CD56+ NK, and CD8+/CD45RA+ Naive Cytotoxic. Zheng *et al.* identify these particular cell-types as demonstrating little sub-structure (Zheng *et al.*, 2017).

UMI count matrices and barcode (cell) metadata are available from the GitHub repository associated with the publication: <https://github.com/10XGenomics/single-cell-3prime-paper/tree/master/pbmc68k_analysis>.

## PBMC5K_Prot

*PBMC5K_Prot* is a dataset of approximately 5 thousand PBMCs sequenced by and available from 10X genomics under the name “5k Peripheral blood mononuclear cells (PBMCs) from a healthy donor with cell surface proteins (v3 chemistry)” and processed under cell ranger version 3.1.0. A panel of 31 surface proteins were sequenced in parallel with the cDNA libraries. We perform unsupervised clustering on the protein abundance estimates to generate pseudo-annotations independently from RNA expression measurements.

## MaltTumor10K

*MaltTumor10K* is a dataset of approximately 10 thousand cells from a MALT tumor sequenced by and available from 10X genomics under the name “10k Cells from a MALT Tumor - Gene Expression and Cell Surface Protein” and processed under cell ranger version 3.0.0. A panel of 17 surface proteins were sequenced in parallel with the cDNA libraries. We perform unsupervised clustering on the protein abundance estimates to generate pseudo-annotations independently from RNA expression measurements.

## MouseBrain

*MouseBrain* is a dataset of approximately 9 thousand mouse brain cells sequenced by and available from 10X genomics under the name “9k Brain Cells from an E18 Mouse” and processed under cell ranger version 1.3.0.

## PBMC68K

*PBMC68K* is a partner dataset to PBMC68K_Pure (Zheng *et al.*, 2017) produced by sequencing approximately 68 thousand PBMCs. In the original paper, pseudo-annotations were generated by computational matching of these cells to the purified lines of PBMC68K_Pure. We, however, treat these as unannotated cells. UMI count matrices are available from 10X genomics under the name “Fresh 68k PBMCs (Donor A)” and processed under cell ranger version 1.1.0.

## EMT

*EMT* is a dataset of 5,004 sequenced MCF10A mammary epithelial cells induced to undergo spontaneous epithelial to mesenchymal transitions (EMTs) through the cellular detection of neighboring unoccupied space (McFaline-Figueroa *et al.*, 2019). This spatial effect allowed the authors to dissect an inner region a-priori expected to be primarily epithelial cells and an outer region a-priori expected to be primarily mesenchymal cells which were then sequenced separately. The authors produced another dataset of cells activated by TGF-β (denoted TGFB in the barcode metadata), but we consider only the first dataset (denoted Mock in the barcode metadata). Included in the initial publication, the authors describe eight gene sets from the Hallmark collection (Liberzon *et al.*, 2015) which they consider to be significantly enriched for activity during EMT. We take this set of terms as a ground truth for assessing power under a range of normalization techniques: ESTROGEN RESPONSE LATE, ESTROGEN RESPONSE EARLY, P53 PATHWAY, KRAS SIGNALING DN, MYC TARGETS V1, MYC TARGETS V2, PI3K AKT MTOR SIGNALING, and EPITHELIAL MESENCHYMAL TRANSITION. The UMI count matrices and barcode metadata are available on GEO under accession number GSE114687.

## Dataset processing

For the un-annotated datasets published by 10X (PBMC5K_Prot, MaltTumor10K, MouseBrain, PBMC68K), the UMI count matrices analyzed in this paper were derived from un-filtered gene-barcode matrices. *emptyDrops* (R package: *DropletUtils*; parameters: lower = 20, niters = 160000, test.ambient = TRUE) was used to differentiate empty droplets from barcodes associated with cells. Cellular barcodes were then defined as those with FDR corrected p-values less than 1e-3. For the datasets with surface protein expression (PBMC5K_Prot and MaltTumor10K), cells were additionally filtered to retain only those with a minimum of 100 protein-specific UMIs. This procedure resulted in datasets with 4978 cells (PBMC5K_Prot), 8670 cells (MaltTumor10K), 3756 cells (MouseBrain), and 77249 cells (PBMC68K). Where applicable, surface protein expression was omitted from the rows of UMI count matrices for downstream analysis and testing.

Pseudo-annotations were generated from the datasets with surface protein expression (PBMC5K_Prot and MaltTumor10K) in a manner similar to the unsupervised clustering of all datasets. Surface protein expression was normalized by the method of median ratio (Anders and Huber, 2010). Note: as all surface proteins are generally expected to have cell-type-specific abundances, this normalization is only expected to equalize counts within cell-types meaning that between cell-type calculations of relative abundance are rendered inapplicable; as our purpose is clustering, this is not a problem. Using the Seurat pipeline, protein expression is reduced to 20 dimensions for PBMC5K_Prot (from 29 distinct proteins) and 15 dimensions for MaltTumor10K (from 17 distinct proteins). Graph based clustering is then performed using *FindNeighbors* and *FindClusters* (additional parameters: algorithm = 3, n.start = 100, n.iter = 100) from the Seurat package.

# S3 Data simulation

## Initial grouping

We generate our simulated datasets from experimentally derived UMIs with the purpose of making our simulations as representative of the characteristics of individual, experimentally derived cells as possible. The first step is to normalize experimental data using Dino (see S4.2) and then perform unsupervised clustering on the normalized data (see S4.4). This results in (relatively) homogenous subsets of cells. Using these cluster annotations, we then generate individual clusters of simulated data from the raw UMI counts from each of these cluster annotations. These simulated clusters, mirroring the cell-type heterogeneity in the original data, are then merged into a test dataset.

## Group filtering

We vary the size of simulated clusters by powers of 2 (40 cells, 80 cells, 160 cells, etc.). To this end, we calculate the largest *k* such that we have *k* calculated clusters with at least *40×2^k^* cells and discard the remaining experimental data. If *k=6*, then we have exactly 6 clusters of experimental data with at least *40×2^6^=2560* cells in each group, and we discard cells from any smaller clusters.

## Cluster pair simulation

The simulated dataset is constructed of pairs of simulated clusters for which the EE and DE genes are known. In the case were *k=6* as above, the simulated datasets then consist of 6 cluster pairs, or 12 simulated clusters total. Within a cluster pair, there is an induced difference in LS between the pairs and DE genes are randomly selected. Between cluster pairs, there may also be systematic differences in LS, but only to the extent that there are systematic differences in the LSs of the experimental cells these cluster pairs are based on.

To construct one cluster pair, an experimental cluster, denoted by *C_k_*, is randomly sampled. Each of the two simulated clusters in the pair will consist of 40 cells if this is the first cluster pair, 80 cells if this is the second cluster pair, and so on increasing by factors of 2. Denote the number of simulated cells in each cluster by *n*. To generate the first *n/2* cells in each cluster, we sample *n* cells from the experimental data. In order of increasing LS, we sum pairs of experimental cells to create *n/2* pseudo-cells with roughly double the LS of either of the cells they are comprised of.

Denote the simulated clusters in the pair by *A* and *B*, each to consist of *n* simulated cells. To induce a difference in LS between *A* and *B*, we sample a LS fold change, *δ_fc_*, from the range *3/*2 to *4*, and for convenience assign *A* to be the group with higher average LS. The first *n/2* cells in each group will be generated by binomial sampling from the *n/2* pseudo-cells, with the induced fold change in LS arising from differences in the binomial probability parameter, *p*. Some algebra shows that the choice of

$$p=0.5\pm\frac{(\delta_{fc}-1)}{2(\delta_{fc}+1)}$$

for *A* and *B* respectively will produce clusters with all EE genes once LS is accounted for under normalization. Specifically, for pseudo-cell *s_j_*, simulated cells *a_j_* and *b_j_* from *A* and *B* respectively are generated as:

$$a_{j}\sim Binom\left( s_{j},p_{+} \right)$$

$$b_{j}\sim Binom(s_{j},p_{-})$$

where *p_+_* and *p_-_* are the two variants of *p* respectively.

However, this approach only generates EE genes. To simulate known DE genes, we subset those genes in *C_k_* with at least 25% non-zeros. From this set of genes, we sample 10 to be induced DE genes, with sampling weighted by the inverse density of log gene expression, calculated simply as the log of the mean UMIs in *C_k_*. As with the fold change in LS, we sample 10 DE fold changes from the range *3/2* to *6*, denoted by *γ_fc,g_* with the subscript *g* indexing the 10 gene-specific DE fold changes. As we do not want all DE genes to be upregulated in *A*, we invert each of the *γ_fc,g_* with probability 0.5. If we now consider the binomial probability, *p*, to be a vector of length equal to the number of genes, and *p_DE_* to denote the subset of elements which are DE after correcting for LS, some similar algebra to the above shows that

$$p_{DE, g}=0.5\pm\frac{(\delta_{fc}\gamma_{fc,g}-1)}{2(\delta_{fc}\gamma_{fc,g}+1)}$$

where this formulation also allows the definition of *a_j_* and *b_j_* to be defined as the same binomial random variable parameterized by *p_+_* or *p_-_*, where *p* now includes information about DE sampling.

Two problems remain to be addressed; that we have only discussed the generation of *n/2* of the cells in each group and that correcting for LS as defined here can induce slight but systematic differential expression in the EE genes. Take, for example, the extreme case where all the DE genes are upregulated in *A* relative to *B* after correcting for LS. In this case, calculating LS from the sum of simulated UMIs within a cell, and correcting for that LS, will induce a slight but consistent down-regulation in the EE genes in *A* relative to *B*. We address this by adding a correction factor to the remaining *n/2* simulated cells in each cluster.

The degree of this induced bias can be simply calculated as the ratio of expected total LS (total meaning summed across cells as well as genes) under the above DE model and the model where all genes are simulated EE. Let *p_+_* be, as above, a vector of binomial probabilities which includes DE information for the simulation of *A* and let *p_EE+_* be a corresponding binomial probability vector for which all genes are EE, that is, suppose all elements of *p_EE+_* are equal to the original, scalar, definition of *p_+_*. Let *C_k-_* denote a vector of gene-wise UMIs, summed across all cells in *C_k_*. Then, the total expected LS for the EE case is *p_EE+_^T^C_k-_* and the degree of bias in the above simulated cells, inducing DE in simulated EE genes, is

$$\alpha_{+bias}=\frac{p_{+}^{T}C_{k-}}{p_{EE+}^{T}C_{k-}}$$

This can be interpreted as implying that normalized EE genes in *A* will, on average, be a factor of *1/α_+bias_* different from what would have been the case had all genes had been simulated as EE. If *α_-bias_=α_+bias_*, then this wouldn’t be a problem, but such is not the case. Unfortunately, it is also the case that *α_-bias_≠1/α_+bias_*, as can be shown by simple counter examples. Therefore, we compute separate corrective factors for *A* and *B* under the principle that expression of EE genes between *A* and *B* should, when averaged across the first *n/2* cells and the second, corrected *n/2* cells demonstrate the desired fold change in LS. This leads to the corrective factor, *c*

$$\left( \frac{c_{+}p_{+}^{T}C_{k-}}{p_{EE}^{T}C_{k-}} \right)^{-1}=1-\left( \frac{1}{\alpha_{+bias}}-1 \right)$$

$$\Rightarrow c_{+}=\frac{1}{2\alpha_{+bias}-1}$$

$$\Rightarrow c_{-}=\frac{1}{2\alpha_{-bias}-1}$$

This then fully defines the simulated cells:

$$a_{j}\sim\left\{ \begin{aligned} Binom\left( s_{j},p_{+} \right), j\leq n/2 \\ Binom\left( s_{j-\frac{n}{2}}, c_{+}p_{+} \right), j>n/2 \end{aligned} \right.$$

$$b_{j}\sim\left\{ \begin{aligned} Binom\left( s_{j},p_{-} \right), j\leq n/2 \\ Binom\left( s_{j-\frac{n}{2}}, c_{-}p_{-} \right), j>n/2 \end{aligned} \right.$$

To complete a simulated dataset, the above steps for generating the cluster pair *A* and *B* are repeated for the remaining experimental clusters, generating a heterogenous samples of simulated data for which pairs of simulated clusters have known EE and DE genes.

# S4 Implementation details

## S4.1 R package versions

BiocParallel (v1.22.0), BiocSingular (v1.4.0), Dino (v0.6.1), DropletUtils (v1.8.0), irlba (v2.3.3), MAST (v1.14.0), Matrix (v1.2-18), matrixStats (0.56.0), mclust (v5.4.6), monocle (v2.16.0), piano (v2.4.0), snowfall (v1.84-6.1), Scran (v1.16.0), sctransform (v0.2.1), Seurat (v3.2.0)

## S4.2 Normalization defaults

**UMI:** reference gene-by-barcode matrix of unique molecular identifiers (UMIs)

**CPM:** rescaling of UMI such that each column (cell or barcode) sums to one million

**CPT:** rescaling of UMI such that each column sums to ten thousand

**Scran:** rescaling of UMI such that each column is divided by the scale factor computed by the scran method from the sizeFactors function in the scran package (Lun *et al.* 2016). Default parameters are used.

**scTrans:** normalized gene-by-barcode matrix output by the vst function in the sctransform package (Hafemeister and Satija, 2019). Default parameters are used excepting: return_cell_attr = TRUE, res_clip_range = c(-50, 50).

**scTransCnt:** corrected UMI count matrix output by the correct_counts function from the sctransform package. Default parameters are used with scTrans and UMI as input.

**Dino:** normalized gene-by-barcode matrix output by the Dino function in the Dino package. Default parameters are used excepting: nCores = 4.

## S4.3 Low-LS vs high-LS QQ plots

The construction of modified Quantile-Quantile plots allows the comparison of normalized expression distributions between low and high-LS cells simultaneously across genes and cell-types. Of particular interest is the determination of whether the distributions of normalized expression for low and high-LS cell are equal. In the case of a homogeneous cell sub-populations, we would expect that these distributions should be equal for well normalized data.

To construct these QQ plots, we proceed for a fixed gene within a cell-type annotation. This fixed gene is sampled from the bottom 90% of expression (calculated as the geometric mean of un-normalized UMIs). The omission of the top 10% of highest expressors is done as these high expressing genes with their correspondingly lower proportion of zeros do result in roughly equivalent normalized distributions between low and high-LS cells for most normalization methods. Given the fixed genes, a grid of quantiles of normalized expression are calculated from the low-LS cells (5%-25% of cells by LS); 2.5% quantile, 5% quantile, 7.5% quantile, and so on. A corresponding grid of quantiles is calculated from the high-LS cells (75%-95% of cells by LS) at the same set of percentiles as the low-LS cells. These two vectors of quantiles then define points on the QQ plot. Unlike typical QQ plots which compare a test dataset to a theoretical distribution, in our modified case we compare two test datasets against each other with the quantiles from the high-LS cells forming the x-coordinates of the points on the plot and the quantiles from the low-LS cells forming the y-coordinates. In this way, if the points lie on the diagonal, we have evidence that the distribution of normalized expression from high-LS cells is similar if not identical to the distribution of normalized expression for the low-LS cells. This is particularly useful as the construction allows this comparison without defining a theoretical distribution to compare the data against.

To extend the comparison across genes and cell-types, corresponding vectors of quantiles are calculated across a random sample of 600 genes in total (bottom 90% of expression), and this calculation is then repeated across cell types. Rather than display the thousands of points resulting from this analysis individually, the modified QQ-plot is converted to a frequency heatmap on the log10 count scale.

## S4.4 Pseudo-time differential expression

Analysis of the EMT dataset in a manner similar to that of the original authors was primarily conducted using Monocle2 (in the monacle package) for pseudo-time ordering and DE testing; piano was used for term enrichment testing. Normalized matrices were log transformed with a +1 pseudo count for variance stabilization excepting scTrans which performs variance stabilization internally to the algorithm. Following variance stabilization normalized matrices were used to create monocle objects for further analysis. For each normalization method, the top 1,000 highest variance genes (after the above-mentioned transformation) were used to perform dimension reduction in the Monocle2 environment which includes construction of a minimum spanning tree using the DDRTree algorithm. This tree was used to estimate pseudo-times for each cell, again using the default Monocle2 pipeline. To root the tree (determine which cell is assigned the pseudo-time of 0), we subset the cells assigned to the branches that contain the default earliest and latest pseudo-time, which denote ends of the longest contiguous differentiation path in well-ordered data. Of these two branches, we denoted the branch with the highest proportion of inner section cells (expected to be primarily epithelial cells) as the root of the tree. Unlike the analysis in the original paper, trees constructed in our application included branches. Because of this, we removed from further analysis cells in any branches not along the main path, defined as the minimum path between the earliest and latest branches (mentioned in the content on rooting). As such, this sub-tree was a non-branching path.

Following computation of pseudo-times and sub-setting of cells, we conducted DE tests, here defined as a change in mean expression over time. Formally, this test is a likelihood ratio test between a natural spline with three degrees of freedom regressed against pseudo-time and an intercept-only model. This test (function differentialGeneTest) is part of the Monocle2 pipeline and was implemented using default parameters.

In their original paper, the authors defined a list of Hallmark gene sets as enriched for expression changes over the epithelial to mesenchymal transition; we consider these as ground truth. Enrichment testing for the Hallmark collection of gene sets was performed using the piano package (runGSA function) using DE test p-values as gene-level statistics and the “tailStrength” statistical test.

## S4.5 Unsupervised clustering analysis

Unsupervised clustering was performed using standard functions in the Seurat package. Normalized matrices were used to create Seurat objects, and were log transformed with a +1 pseudo count for variance stabilization excepting scTrans which performs variance stabilization internally to the algorithm. The top 1,000 highest variance genes were subset to perform dimension reduction using approximate PCA down to 20 dimensions. Per-cell cluster memberships were identified using graph-based clustering using the FindClusters function in Seurat with default parameters excepting: algorithm = 3, n.start = 50, n.iter = 50.

To assess the accuracy of unsupervised techniques based on different normalization pipelines, the Adjusted Rand Index (ARI) was calculated between the calculated cluster memberships and cell-type annotations. When datasets comprise greater than 25,000 cells, a random sample of 25,000 cells was subset, normalized, and sent through the clustering analysis pipeline. Calculated clusters for the subset of cells were then compared against the corresponding subset of cell-type annotations.

## S4.6 Down sampled clustering analysis

Clustering analysis was also performed in an environment of exaggerated differences in LS to highlight the impact of differences in LS, even within otherwise homogenous cell types. In this analysis, half of the original cells (up to 25,000 as described in S4.5) from each dataset were randomly selected and down sampled to 25% of their original LS. The full set of cells, both down sampled and unmodified, were then passed through the analysis pipeline from S4.5.

# S5 Supplemental references

Bacher,R. *et al.* (2017) SCnorm: Robust normalization of single-cell RNA-seq data. *Nat. Methods*, **14**, 584–586.

Branham, R. L.,J. (1982) Alternatives to least squares. *Astron. J.*, **87**, 928.

Chen,S.X. (2000) Probability Density Function Estimation Using Gamma Kernels. *Ann. Inst. Stat. Math.*, **52**, 471–480.

Hafemeister,C. and Satija,R. (2019) Normalization and variance stabilization of single-cell RNA-seq data using regularized negative binomial regression. *Genome Biol.*, **20**, 296.

Jamshidian,M. and Jennrich,R.I. (1997) Acceleration of the EM Algorithm by using Quasi-Newton Methods. *J. R. Stat. Soc. Ser. B (Statistical Methodol.*, **59**, 569–587.

Liberzon,A. *et al.* (2015) The Molecular Signatures Database Hallmark Gene Set Collection. *Cell Syst.*, **1**, 417–425.

Lun,A.T.L. *et al.* (2016) Pooling across cells to normalize single-cell RNA sequencing data with many zero counts. *Genome Biol.*, **17**, 1–14.

Van Der Maaten,L. (2014) Accelerating t-SNE using tree-based algorithms. *J. Mach. Learn. Res.*, **15**, 3221–3245.

van der Maaten,L. and Hinton,G. (2008) Visualizing High-Dimensional Data Using t-SNE. *J. Mach. Learn. Res.*, **9**, 2579–2605.

McFaline-Figueroa,J.L. *et al.* (2019) A pooled single-cell genetic screen identifies regulatory checkpoints in the continuum of the epithelial-to-mesenchymal transition. *Nat. Genet.*, **51**, 1389–1398.

Powell,J.L. (1986) Censored regression quantiles. *J. Econom.*, **32**, 143–155.

Powell,J.L. (1984) Least absolute deviations estimation for the censored regression model. *J. Econom.*, **25**, 303–325.

Zheng,G.X.Y. *et al.* (2017) Massively parallel digital transcriptional profiling of single cells. *Nat. Commun.*, **8**, 1–12.


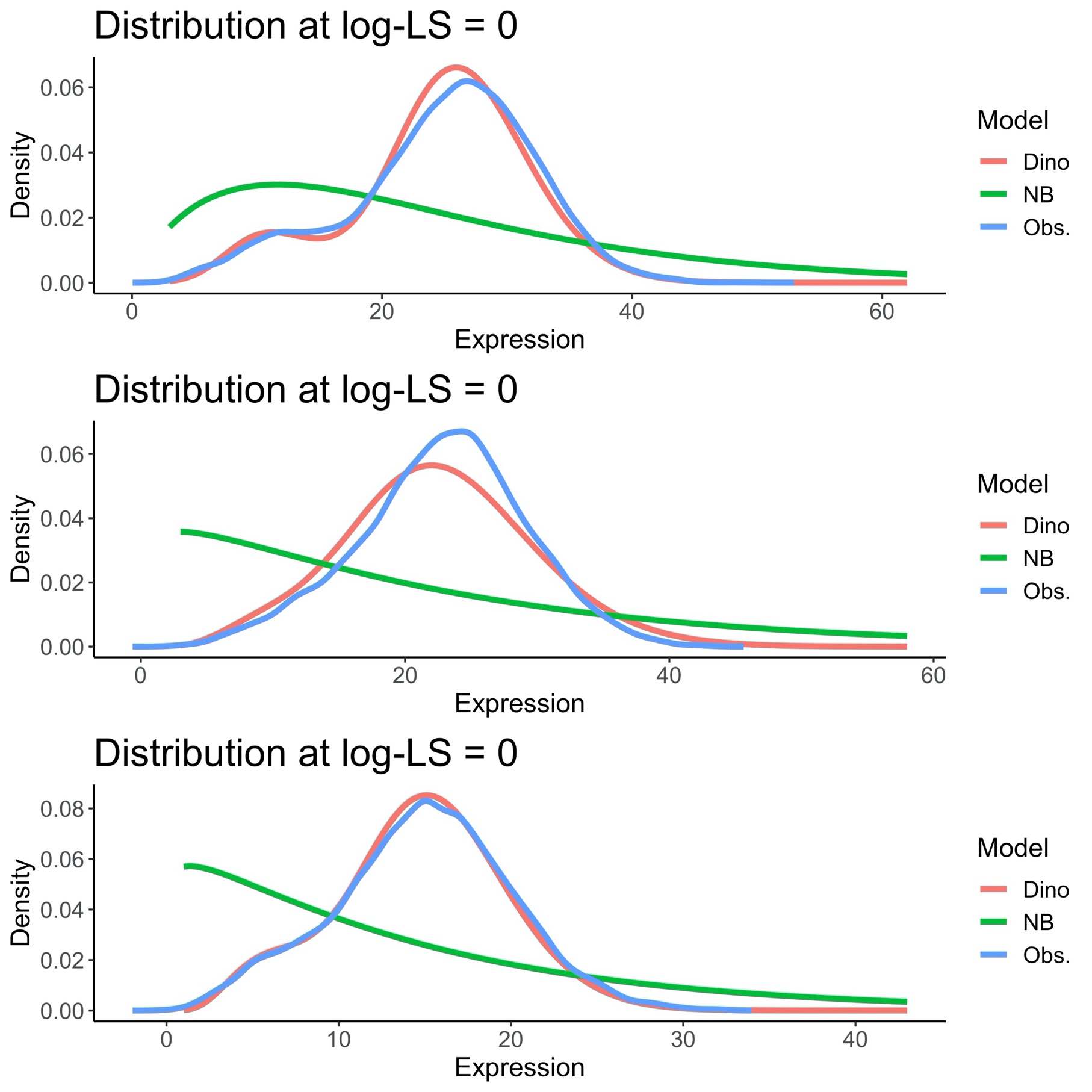


Supplemental Figure S1: **A single Negative Binomial distribution fails to capture the heterogeneity of observed expression.** For each of three different genes (RPL13, RPL10, RPS4X, top to bottom) from the PBMC68K_Pure dataset we plotted the empirical density of observed UMI counts (blue) from cells with scaled log-LS between -0.5 and 0.5. These curves were overlayed by the distribution (at log-LS=0) implied by the fitted parameters from a single Negative Binomial GLM (green) and the prior distribution estimated by Dino (red) used for resampling normalized expression.


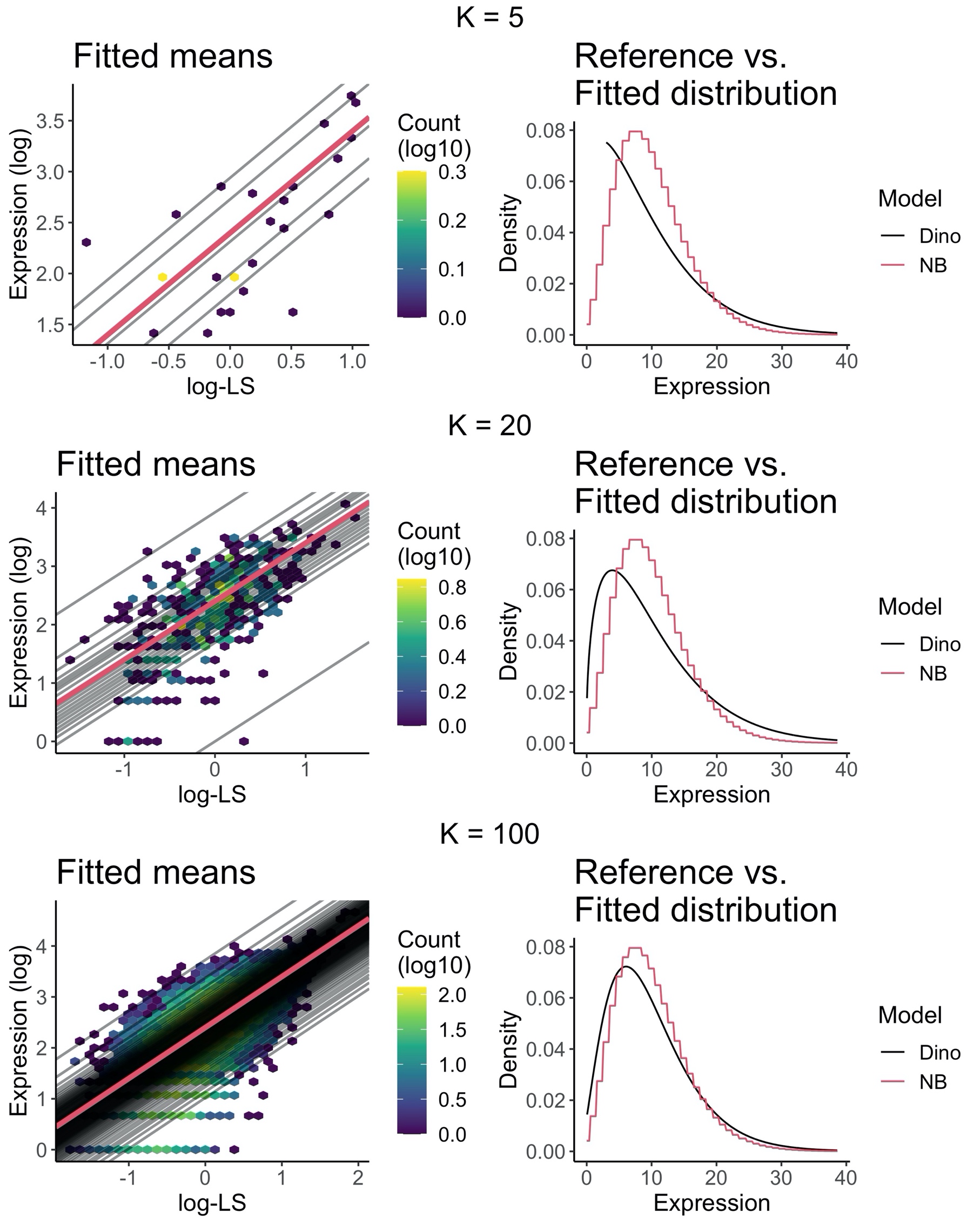

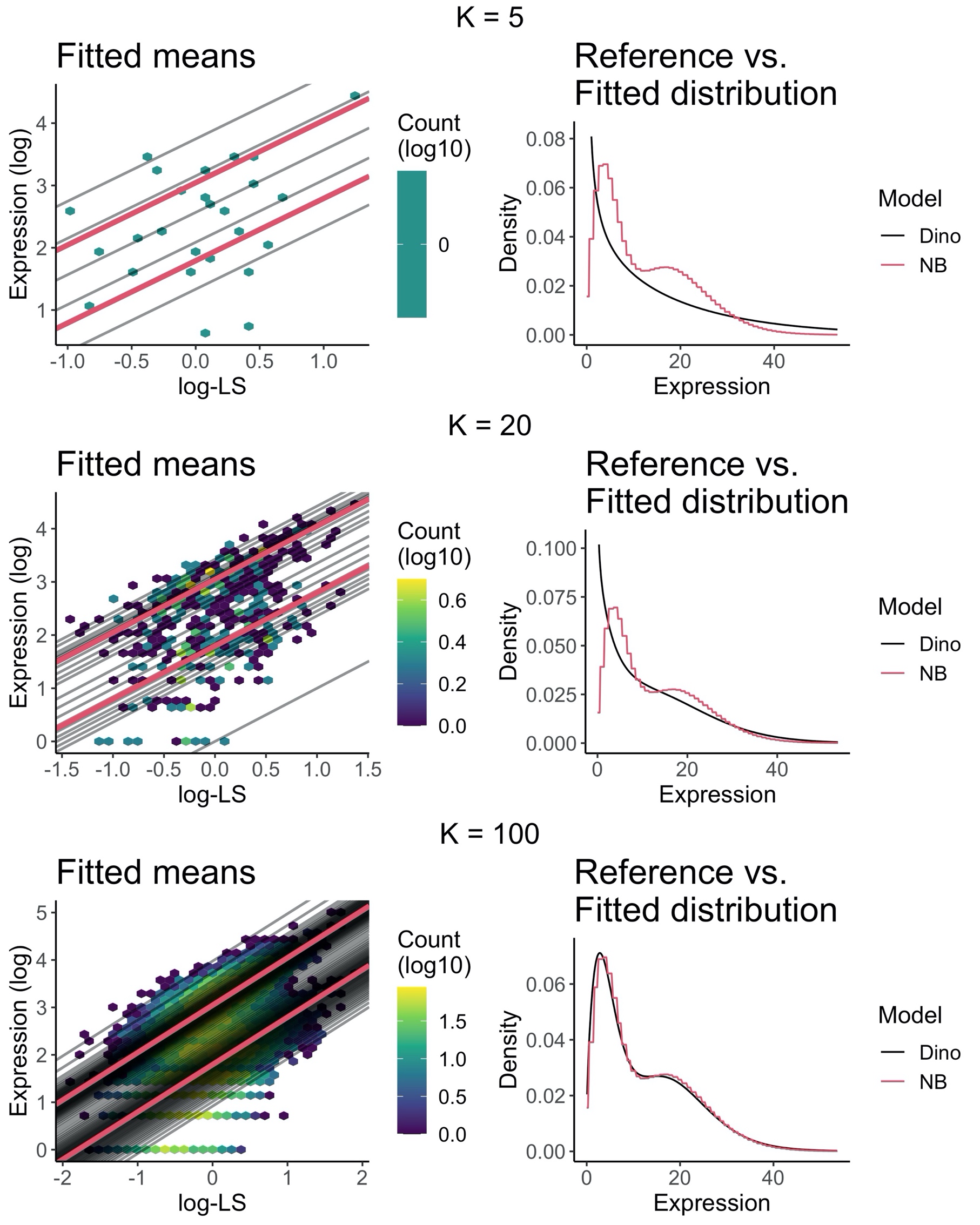


Supplemental Figure S2: **Larger values of *K* improve model fit for unimodal distributions.** We simulated UMI count data under a unimodal Negative Binomial distribution. Each row of the figure indicates a new simulation with sequentially larger values of *K* for the Dino fit. The background of the left column plots the simulated UMI counts as a heatmap (log expression against log library size). Overlaying this heatmap are the mean trend lines fitted from the Dino mixture model (gray) and the true mean of the sampling distribution (red). The right column plots the true distribution of the simulated data at LS=0 (red) and the Dino estimate of the prior distribution which is subsequently used to generate normalized expression values. In practice *K* is chosen algorithmically for each gene, and so small values of *K* relate to correspondingly fewer data points, hence the sparsity of the heatmap of simulated data for *K=5*.

Supplemental Figure S3: **Larger values of *K* improve model fit for multimodal distributions.** We simulated equal numbers of UMI counts from a mixture of two NB distributions, each with different means and dispersion parameters. Each row of the figure indicates a new simulation with sequentially larger values of *K* for the Dino fit. The background of the left column plots the simulated UMI counts as a heatmap (log expression against log library size). Overlaying this heatmap are the mean trend lines fitted from the Dino mixture model (gray) and the true means of the sampling distribution (red). The right column plots the true distribution of the simulated data at LS=0 (red) and the Dino estimate of the prior distribution which is subsequently used to generate normalized expression values. In practice *K* is chosen algorithmically for each gene, and so small values of *K* relate to correspondingly fewer data points, hence the sparsity of the heatmap of simulated data for *K=5*.


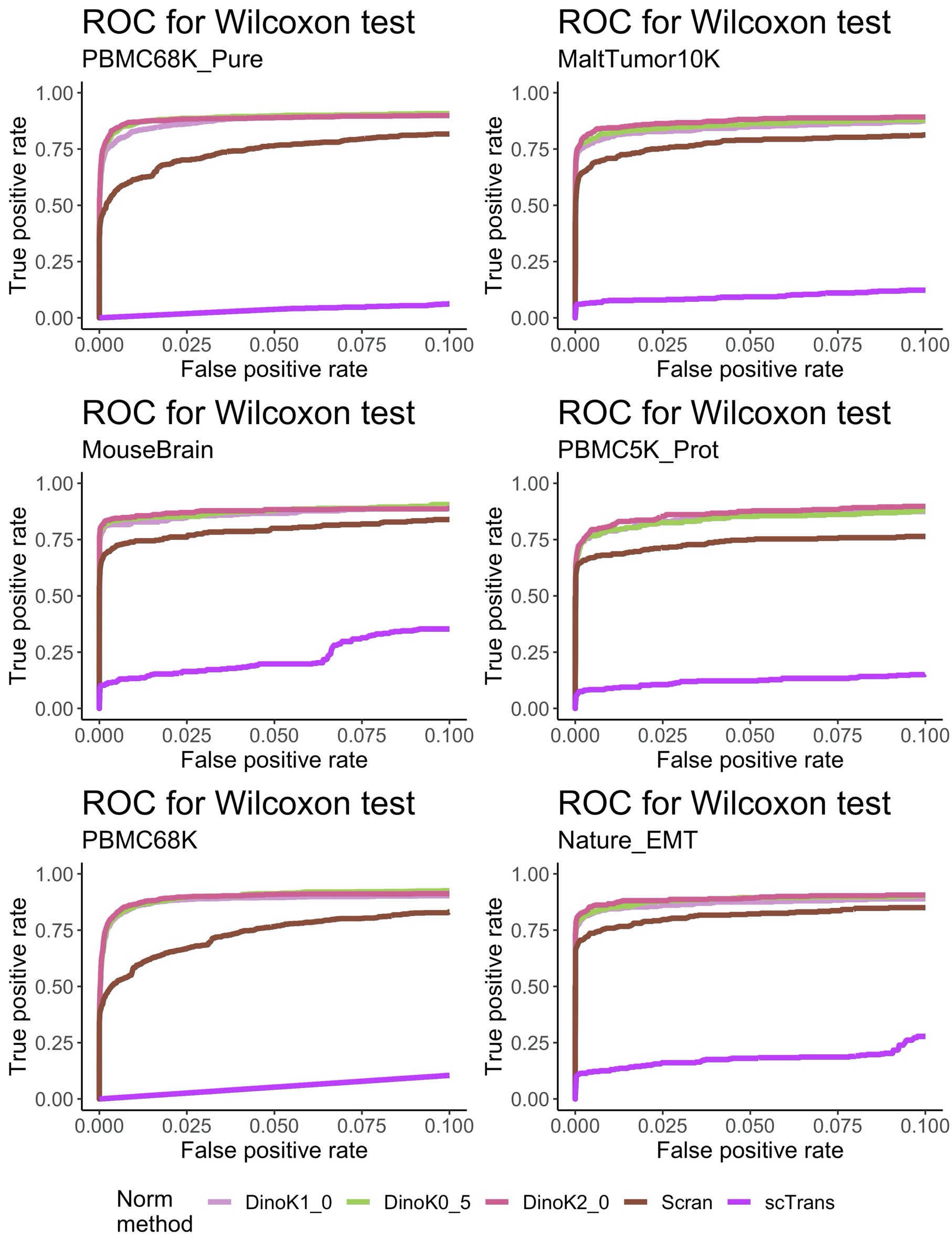


Supplemental Figure S4: **Dino is robust to variations in the gene-specific choice of *K*.** Simulated data based on each of the considered datasets were normalized using each method. ROC curves colored by normalization method define the relationship between average TPR (Power) and average FPR for a Wilcoxon rank sum test, where the average is calculated across 12 simulations from each dataset. DinoK1_0 denotes the default Dino algorithm. DinoK0_5 and DinoK2_0 denote variations with half and double the default per-gene number of mixture components respectively (and 50/200 as maximum allowed values of K).


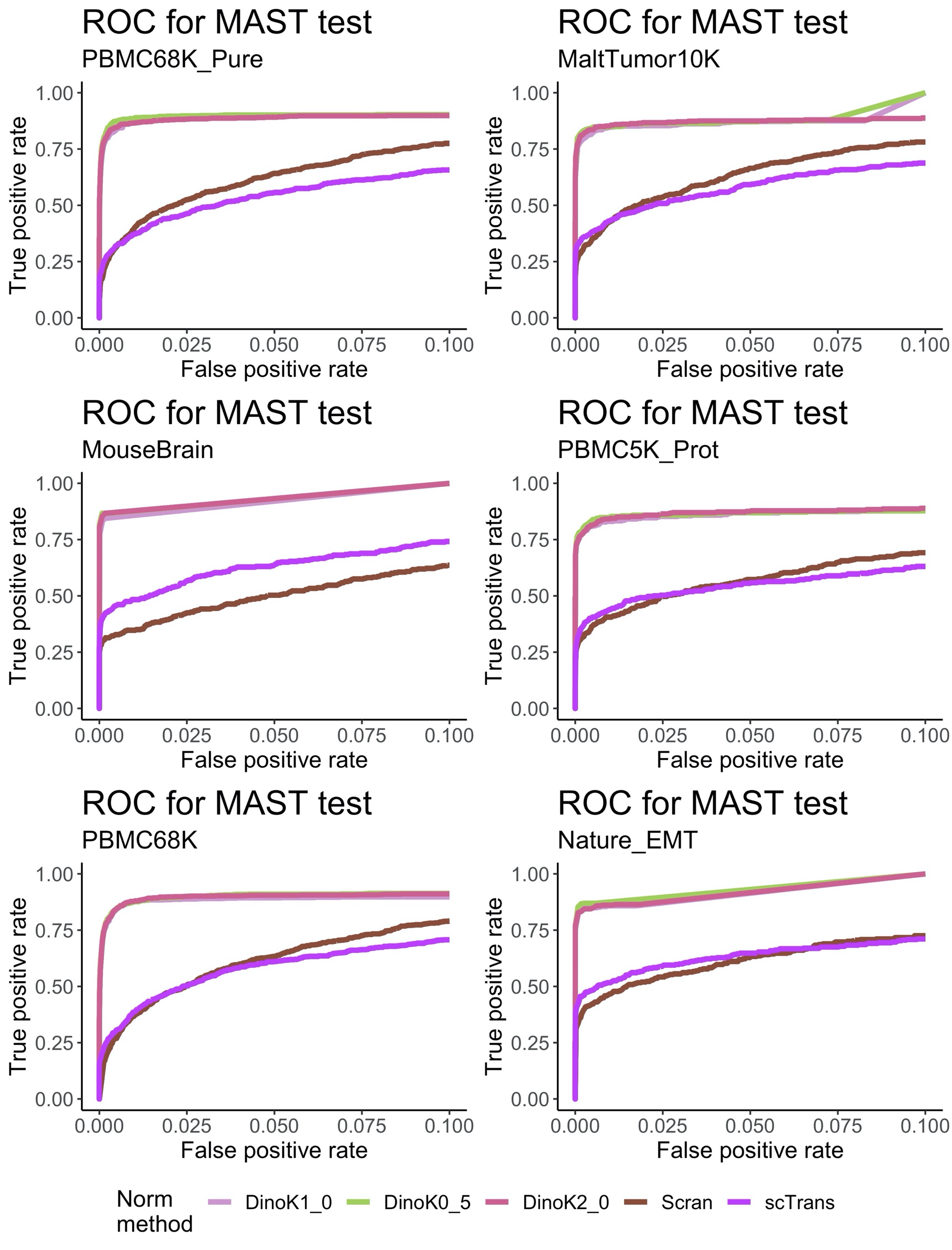


Supplemental Figure S5: **Dino is robust to variations in the gene-specific choice of *K*.** Simulated data based on each of the considered datasets were normalized using each method. ROC curves colored by normalization method define the relationship between average TPR (Power) and average FPR for a MAST test, where the average is calculated across 12 simulations from each dataset. DinoK1_0 denotes the default Dino algorithm. DinoK0_5 and DinoK2_0 denote variations with half and double the default per-gene number of mixture components respectively (and 50/200 as maximum allowed values of K).


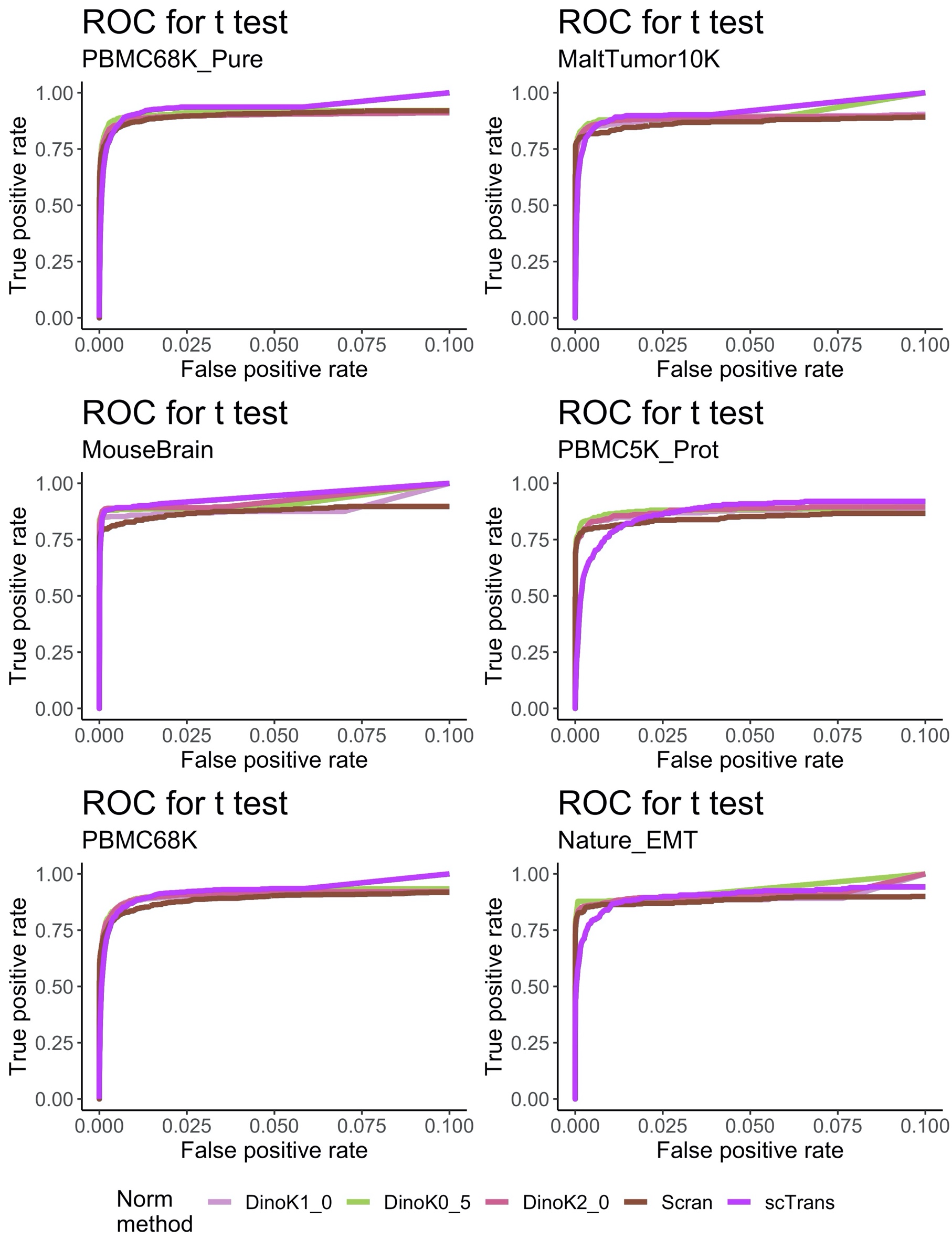


Supplemental Figure S6: **Dino is robust to variations in the gene-specific choice of *K*.** Simulated data based on each of the considered datasets were normalized using each method. ROC curves colored by normalization method define the relationship between average TPR (Power) and average FPR for a t-test, where the average is calculated across 12 simulations from each dataset. DinoK1_0 denotes the default Dino algorithm. DinoK0_5 and DinoK2_0 denote variations with half and double the default per-gene number of mixture components respectively (and 50/200 as maximum allowed values of K).


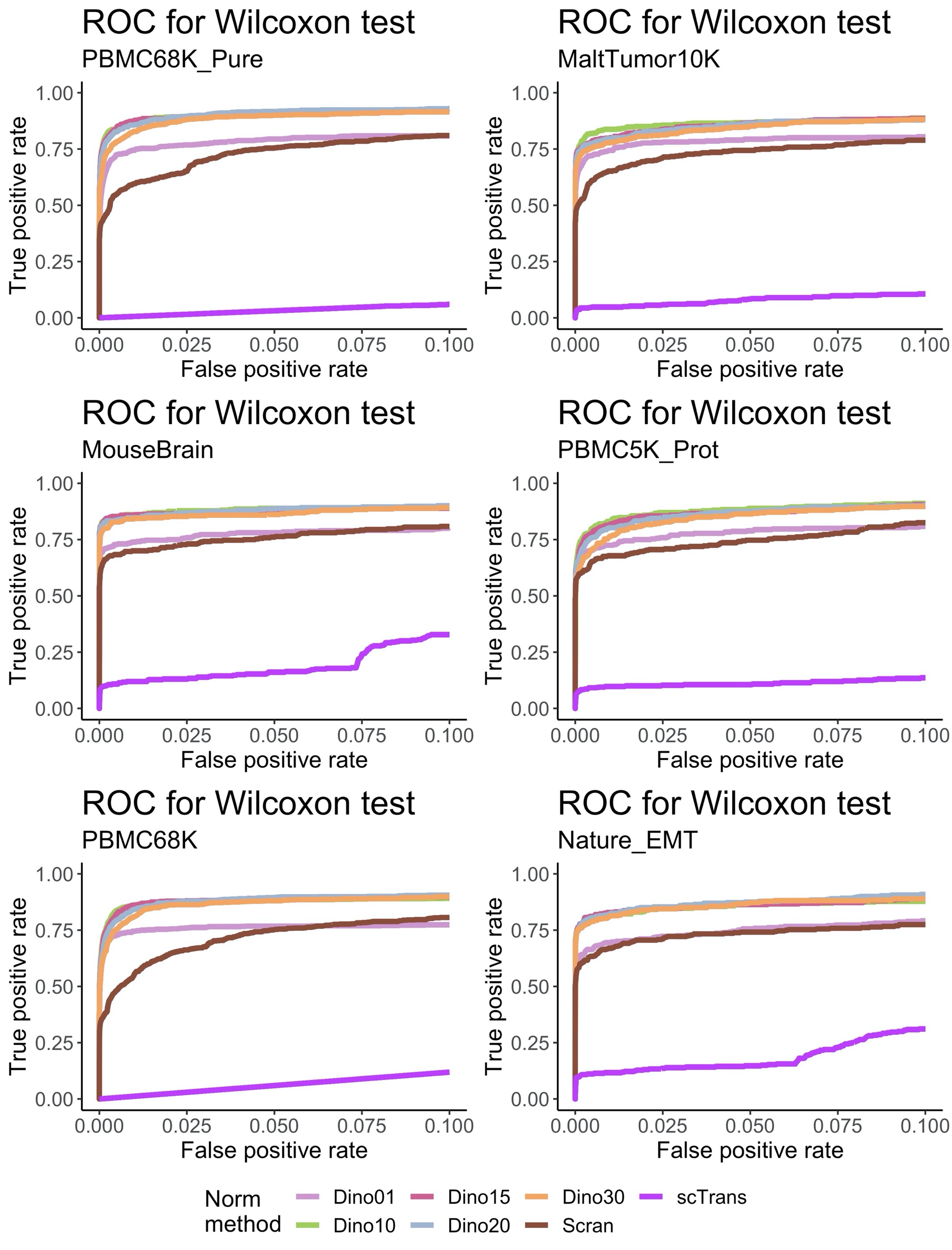


Supplemental Figure S7: **Dino is robust to the choice of the concentration parameter.** Simulated data based on each of the considered datasets were normalized using each method. ROC curves colored by normalization method define the relationship between average TPR (Power) and average FPR for a Wilcoxon rank sum test, where the average is calculated across 12 simulations from each dataset. The concentration parameter (default 15) used for each run of Dino is indicated in the numeric suffix.


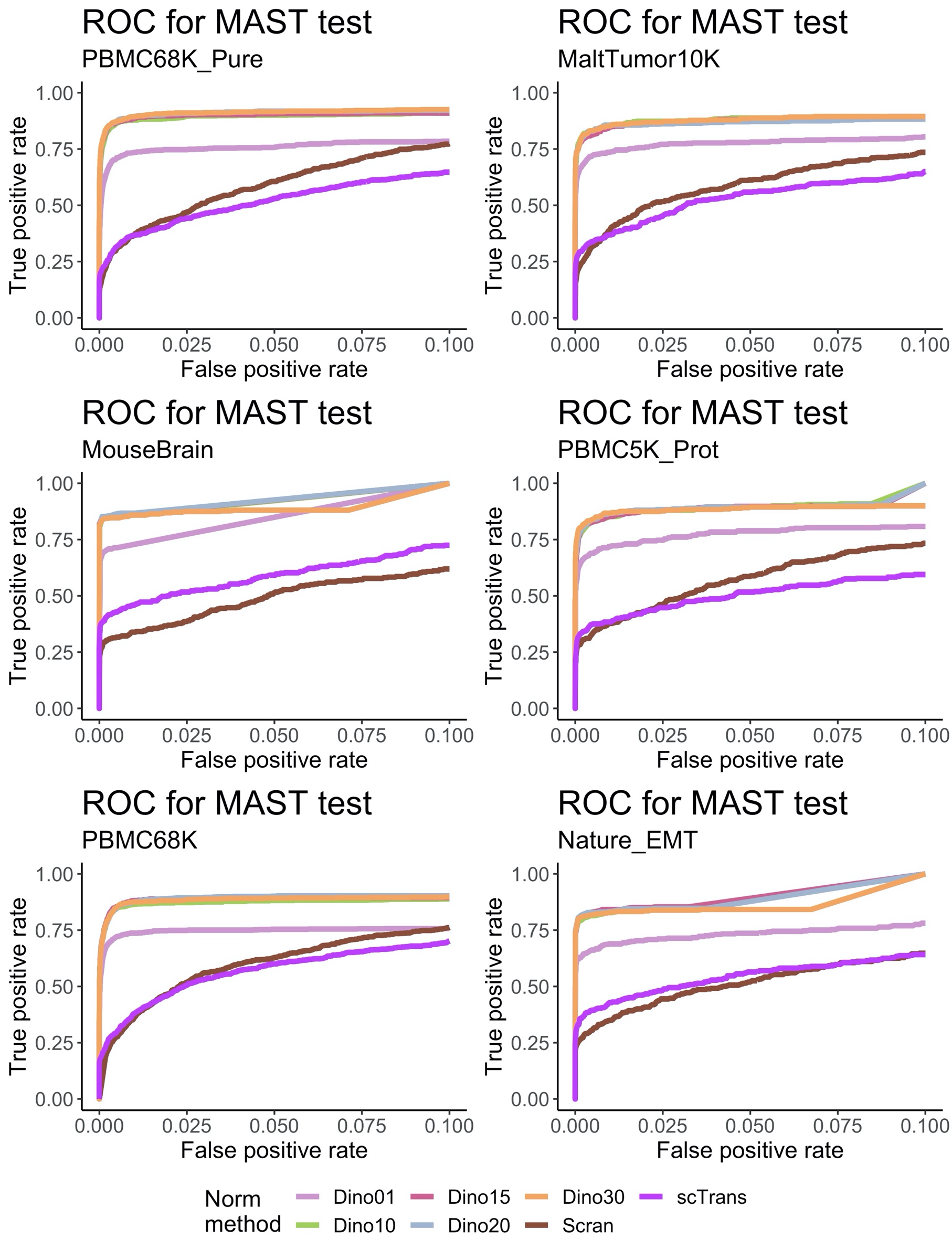


Supplemental Figure S8: **Dino is robust to the choice of the concentration parameter.** Simulated data based on each of the considered datasets were normalized using each method. ROC curves colored by normalization method define the relationship between average TPR (Power) and average FPR for a MAST test, where the average is calculated across 12 simulations from each dataset. The concentration parameter (default 15) used for each run of Dino is indicated in the numeric suffix.


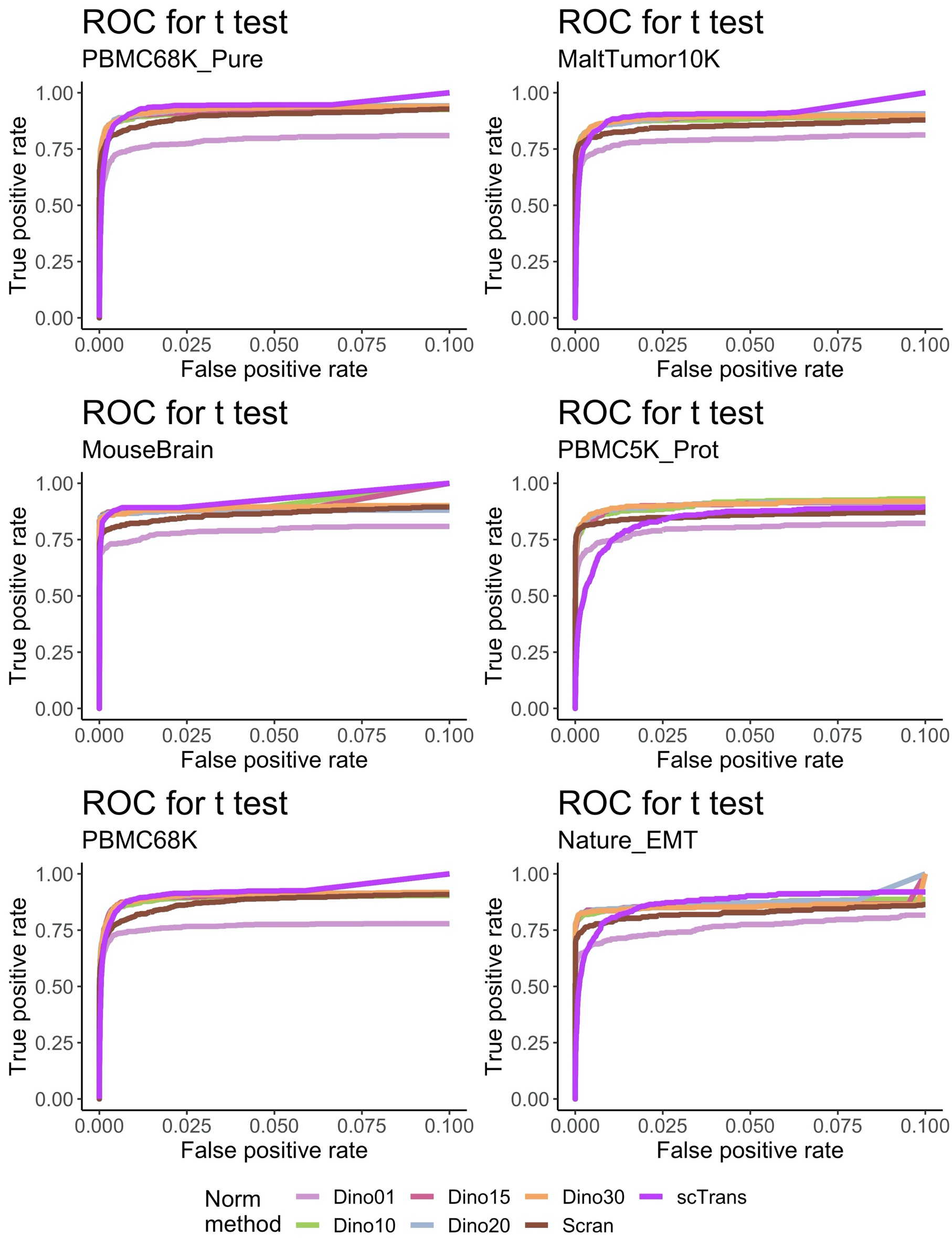


Supplemental Figure S9: **Dino is robust to the choice of the concentration parameter.** Simulated data based on each of the considered datasets were normalized using each method. ROC curves colored by normalization method define the relationship between average TPR (Power) and average FPR for a t-test, where the average is calculated across 12 simulations from each dataset. The concentration parameter (default 15) used for each run of Dino is indicated in the numeric suffix.


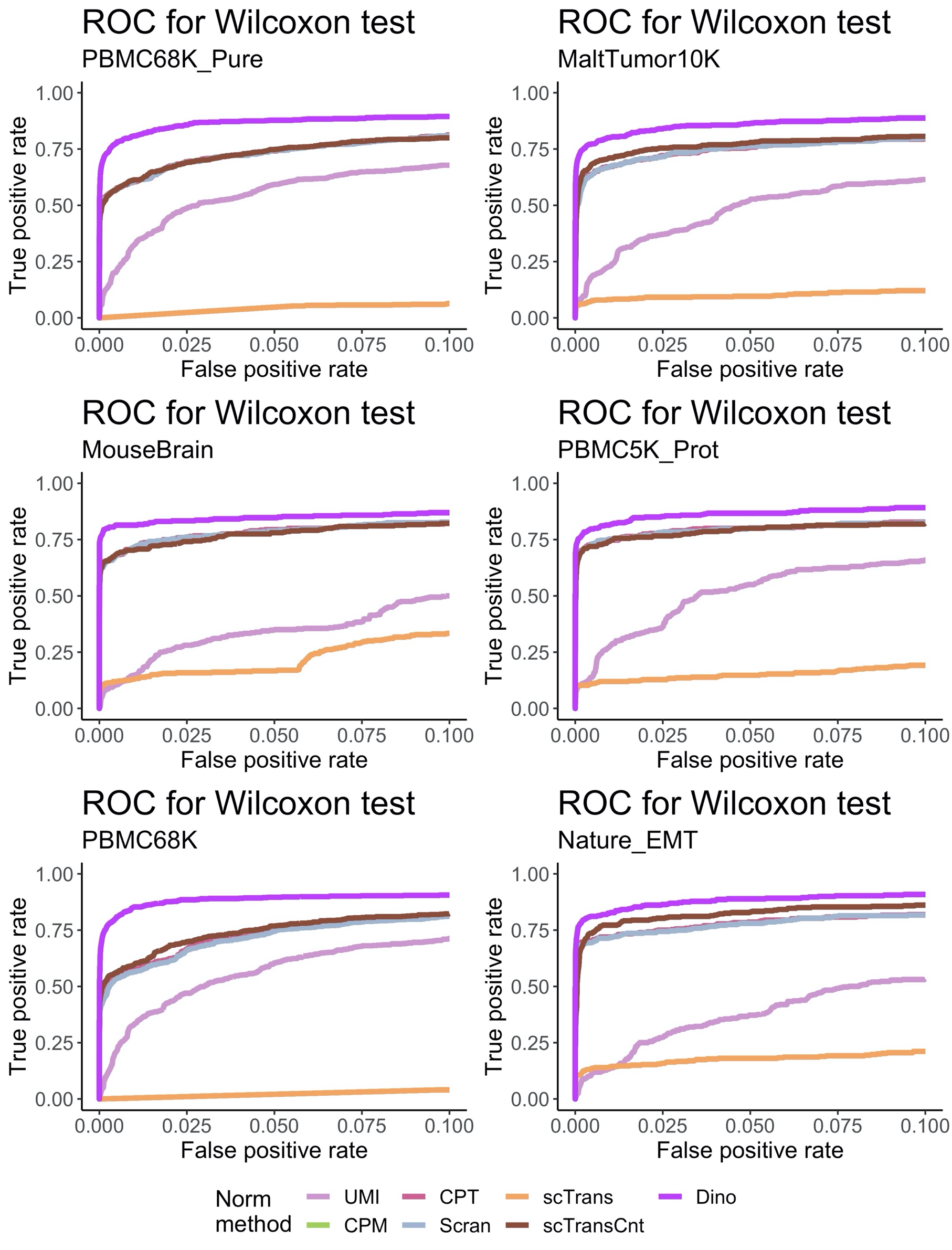


Supplemental Figure S10: **Relative Power/FPR between normalization methods is not significantly affected by the choice in pre-clustering method.** Simulated data based on each of the considered datasets were normalized using each method. ROC curves colored by normalization method define the relationship between average TPR (Power) and average FPR for a Wilcoxon rank sum test, where the average is calculated across 12 simulations from each dataset. Pre-clustering was performed on Scran normalized data for all datasets excepting PBMC68K_Pure where cell annotations were used to define clusters.


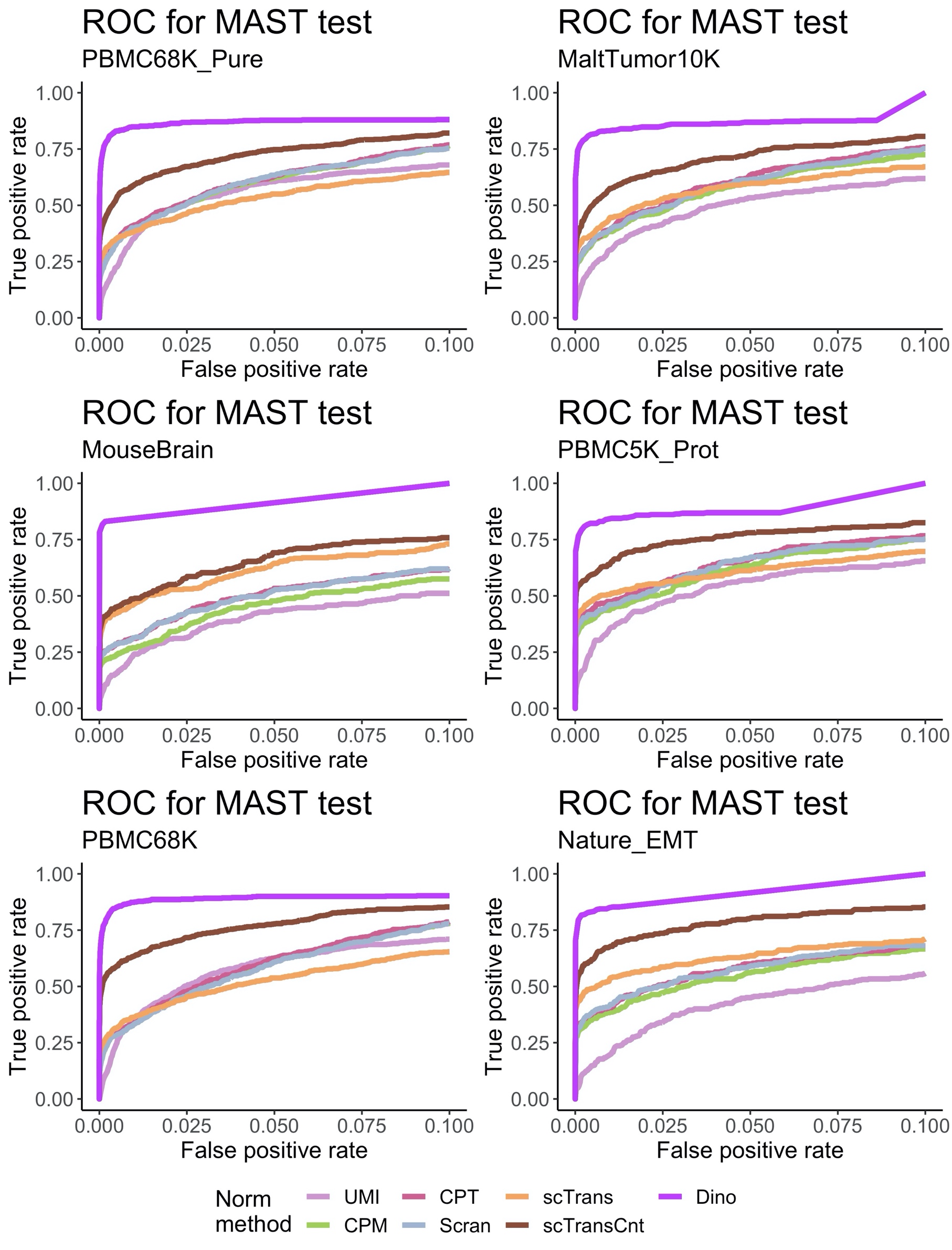


Supplemental Figure S11: **Relative Power/FPR between normalization methods is not significantly affected by the choice in pre-clustering method.** Simulated data based on each of the considered datasets were normalized using each method. ROC curves colored by normalization method define the relationship between average TPR (Power) and average FPR for a MAST test, where the average is calculated across 12 simulations from each dataset. Pre-clustering was performed on Scran normalized data for all datasets excepting PBMC68K_Pure where cell annotations were used to define clusters.


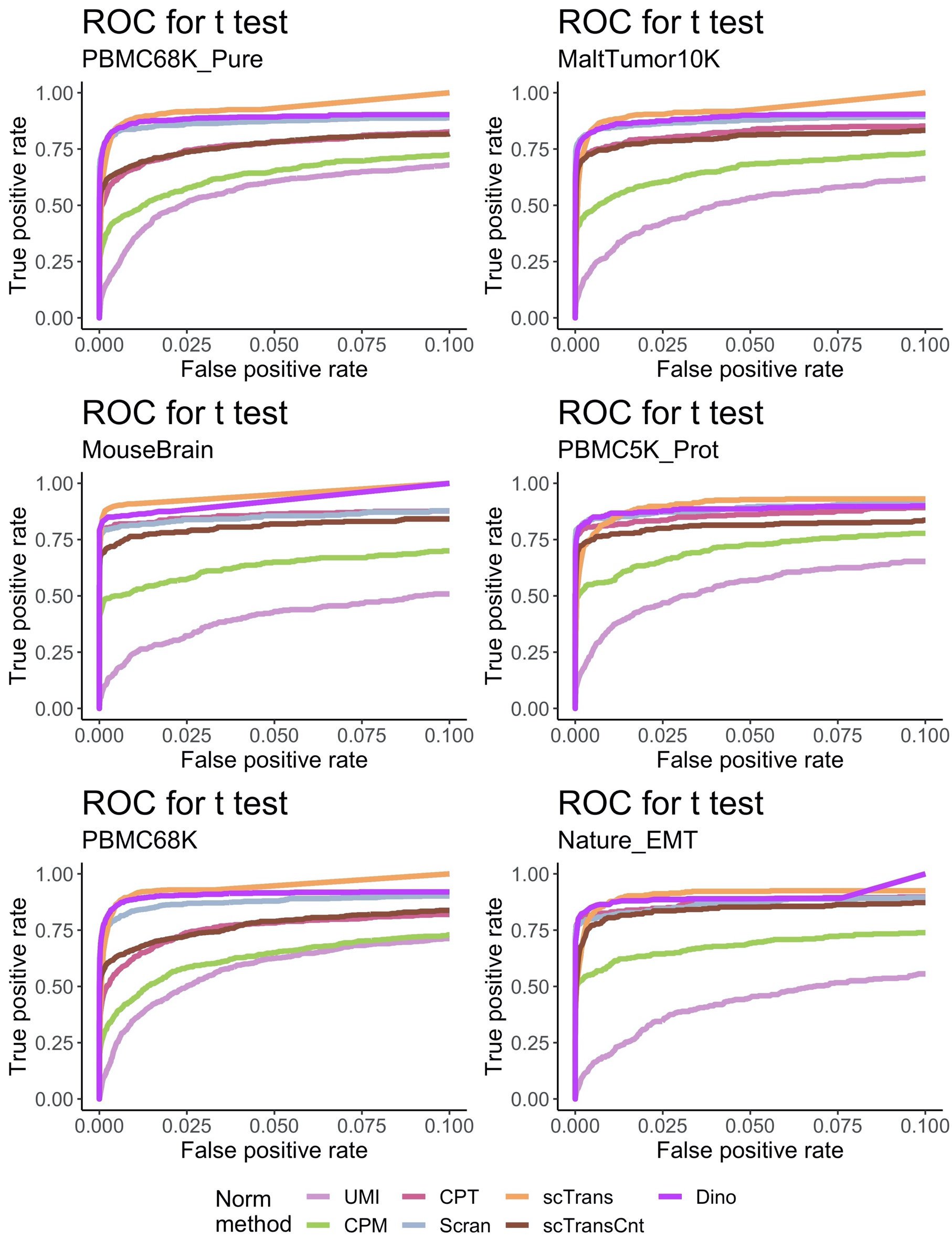


Supplemental Figure S12: **Relative Power/FPR between normalization methods is not significantly affected by the choice in pre-clustering method.** Simulated data based on each of the considered datasets were normalized using each method. ROC curves colored by normalization method define the relationship between average TPR (Power) and average FPR for a t-test, where the average is calculated across 12 simulations from each dataset. Pre-clustering was performed on Scran normalized data for all datasets excepting PBMC68K_Pure where cell annotations were used to define clusters.


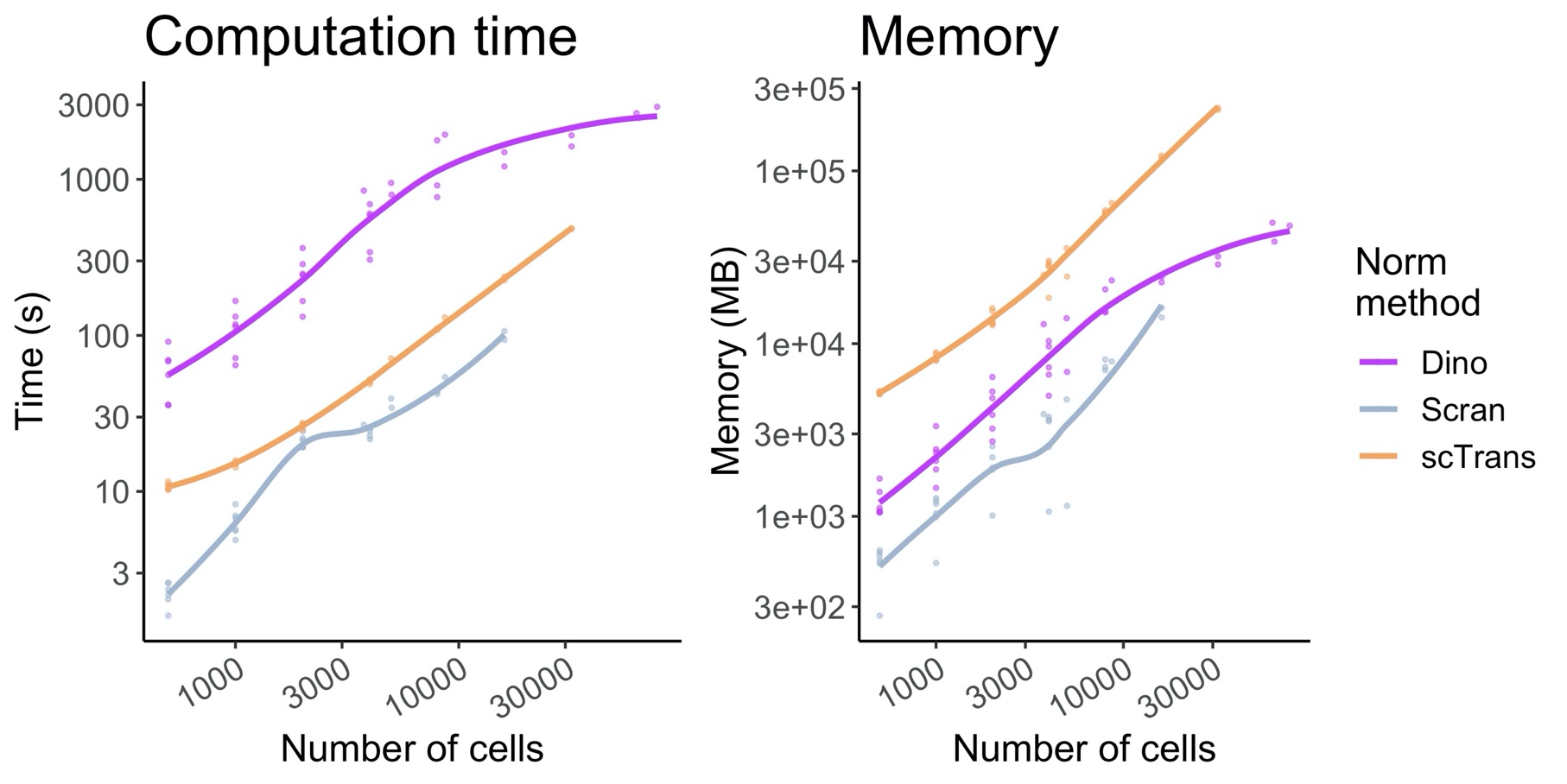


Supplemental Figure S13: **Computational time and memory requirements.** Estimations of resource intensity were conducted on a 2017 MacBook Pro using a 3.1 GHz quad-core CPU and 16 GB of RAM. Memory usage was estimated as the maximum allocated memory as measured by the *Rprof* function and so does not include released memory during the normalization run. The plotted trend lines represent averaged results across samples of the six test datasets described in the manuscript. The trend lines for scTransform and Scran end when the demanded memory resources exceeded those available.


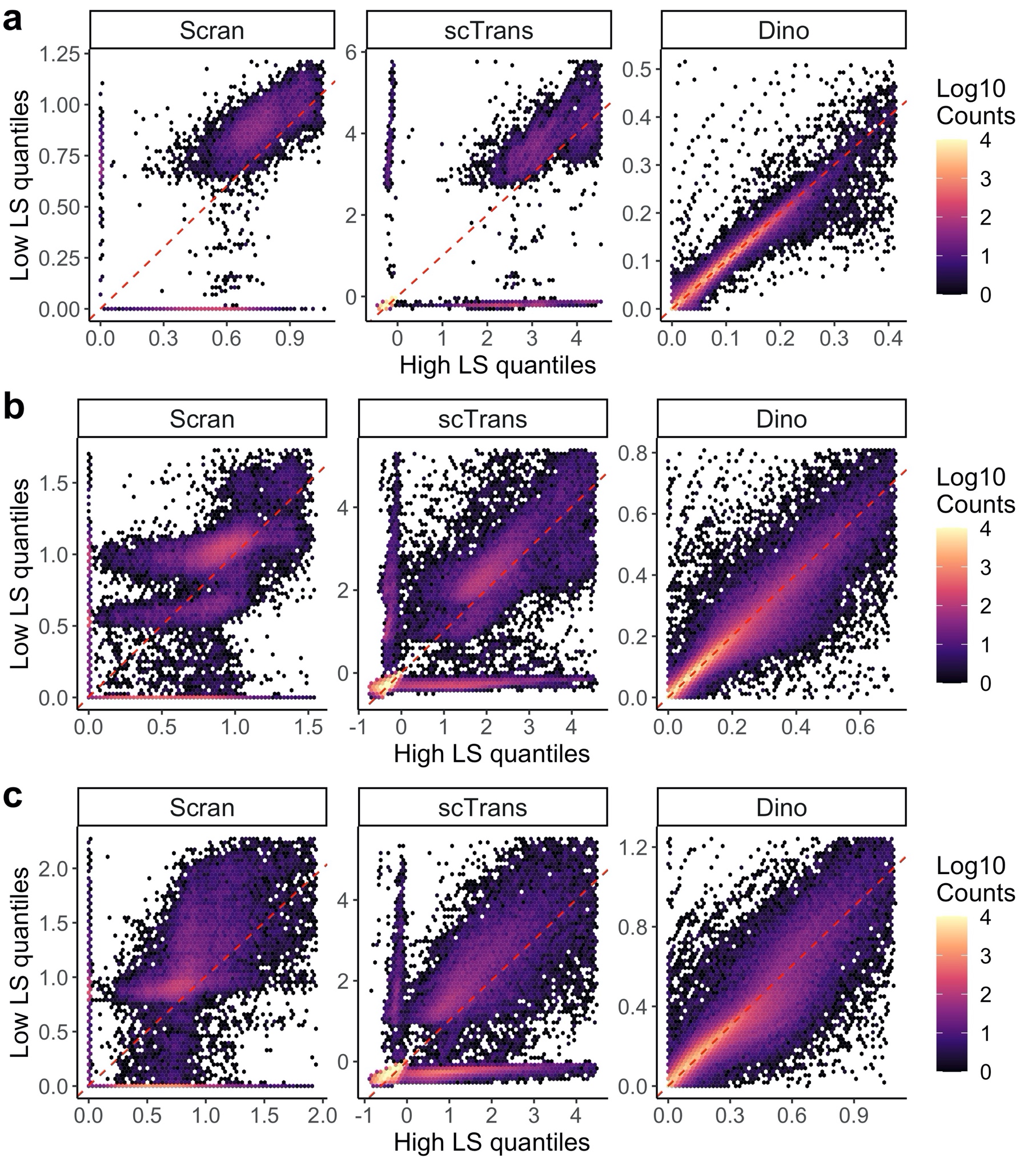


Supplemental Figure S14: **Evaluation of normalized expression distributions across genes and cell types.** Quantile-quantile frequency plots (S4.3 Section) comparing expression quantiles in the high-LS (75%-95% of LS; x-coordinate) and low-LS (5%-25% of LS; y-coordinate) cells across genes and cell-type annotations are shown for the PBMC68K_Pure dataset (a), the MaltTumor10K dataset (b), and the PBMC5K_Prot dataset (c). Colors are log10-scale counts and datapoints in a small neighborhood of zero are omitted to improve visualization.


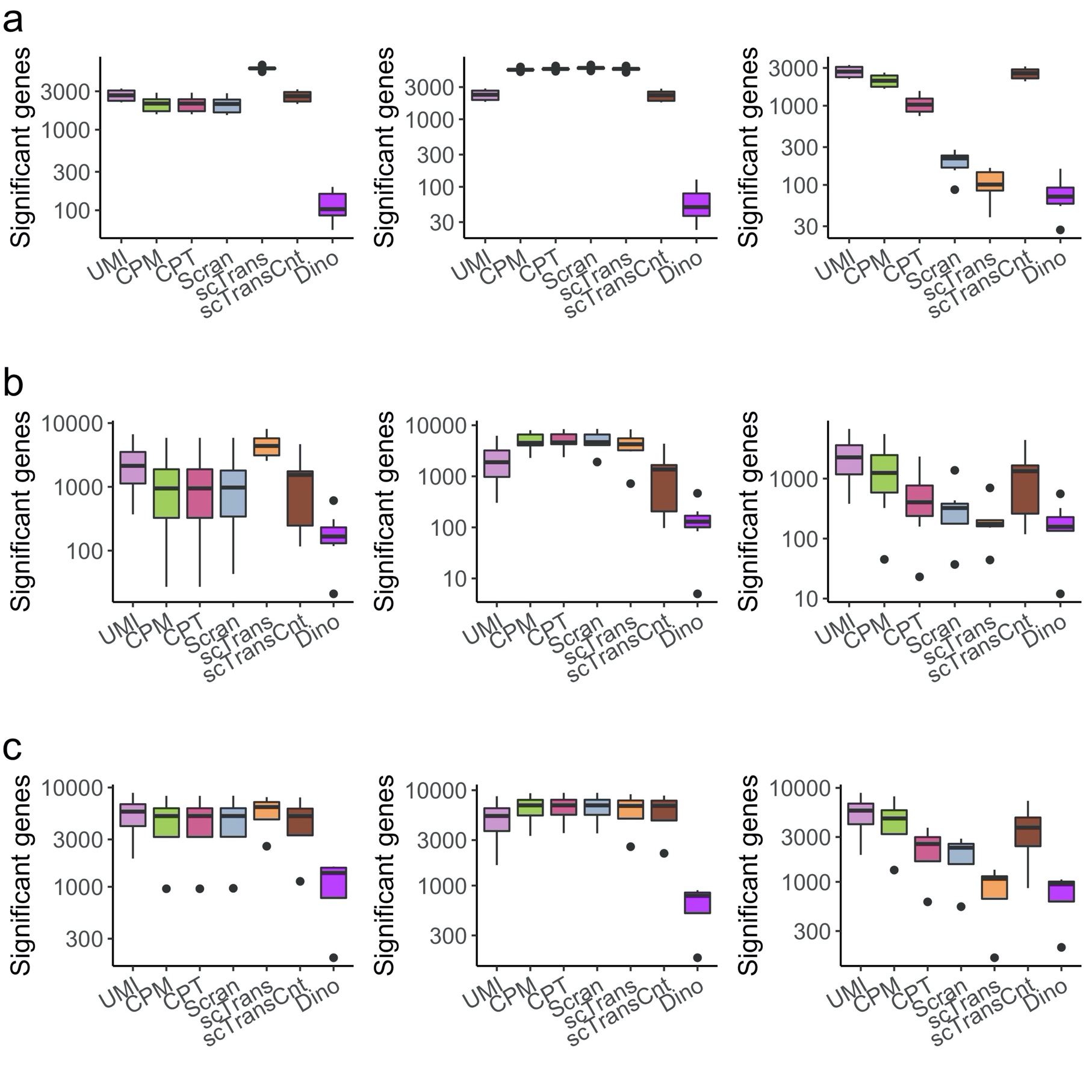


Supplemental Figure S15: **The effects of normalization on downstream DE testing and false positives.** Expression data were normalized and genes were tested for DE using a Wilcoxon rank sum test (left), MAST test (center), and t-test (right) between low-LS and high-LS cells (5%-25% and 75%-95% of LS across cells) within cell-type annotations / pseudo-annotations as applicable. Box plots show the numbers of significant genes in the PBMC68K_Pure dataset (a), the MaltTumor10K dataset (b), and the PBMC5K_Prot dataset (c). Given that cells only differ in LS, DE identifications are expected to be false positives.


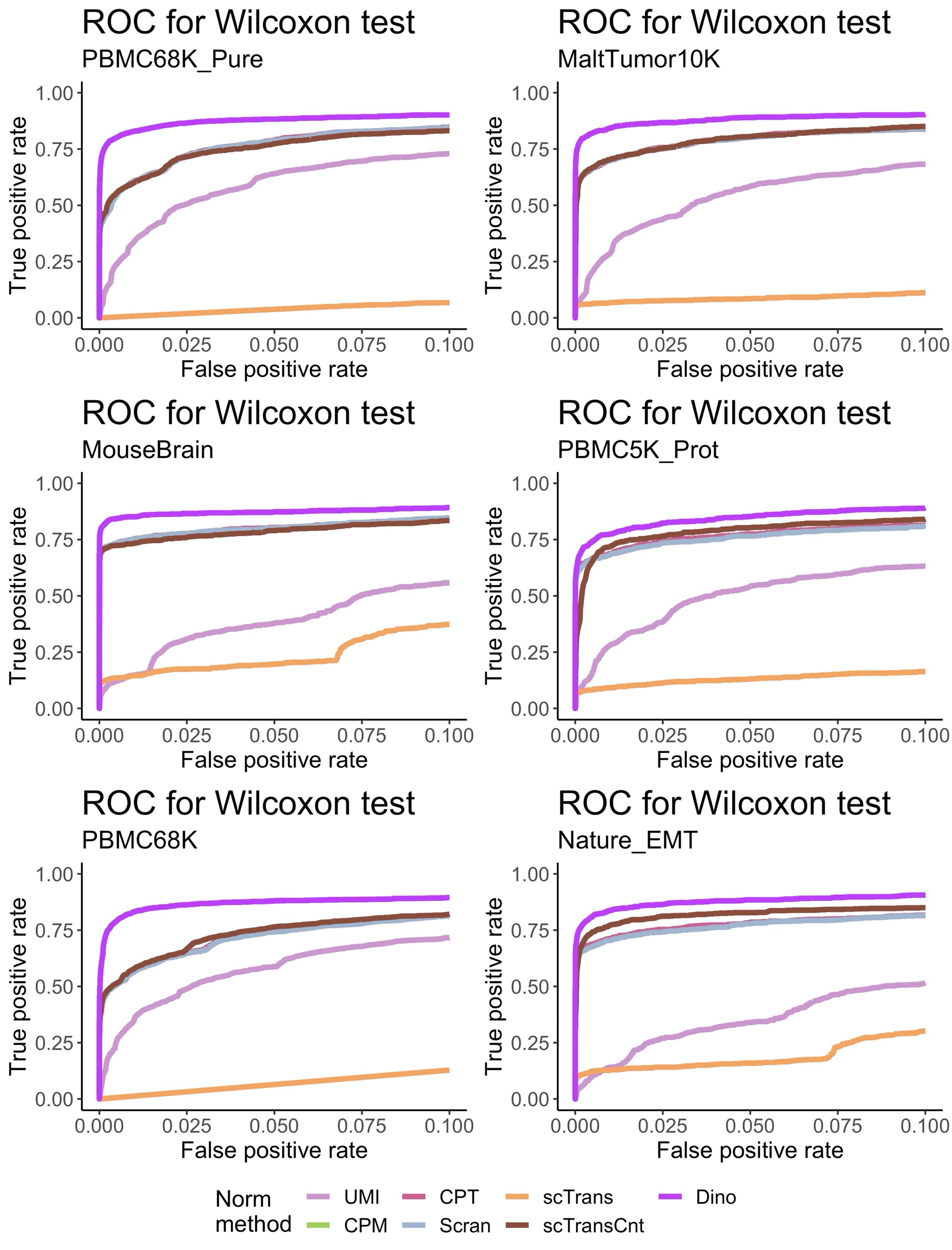


Supplemental Figure S16: **The effects of normalization on downstream DE analysis.** Simulated data based on each of the considered datasets were normalized using each method. ROC curves colored by normalization method define the relationship between average TPR (Power) and average FPR for a Wilcoxon rank sum test, where the average is calculated across 30 simulations from each dataset.


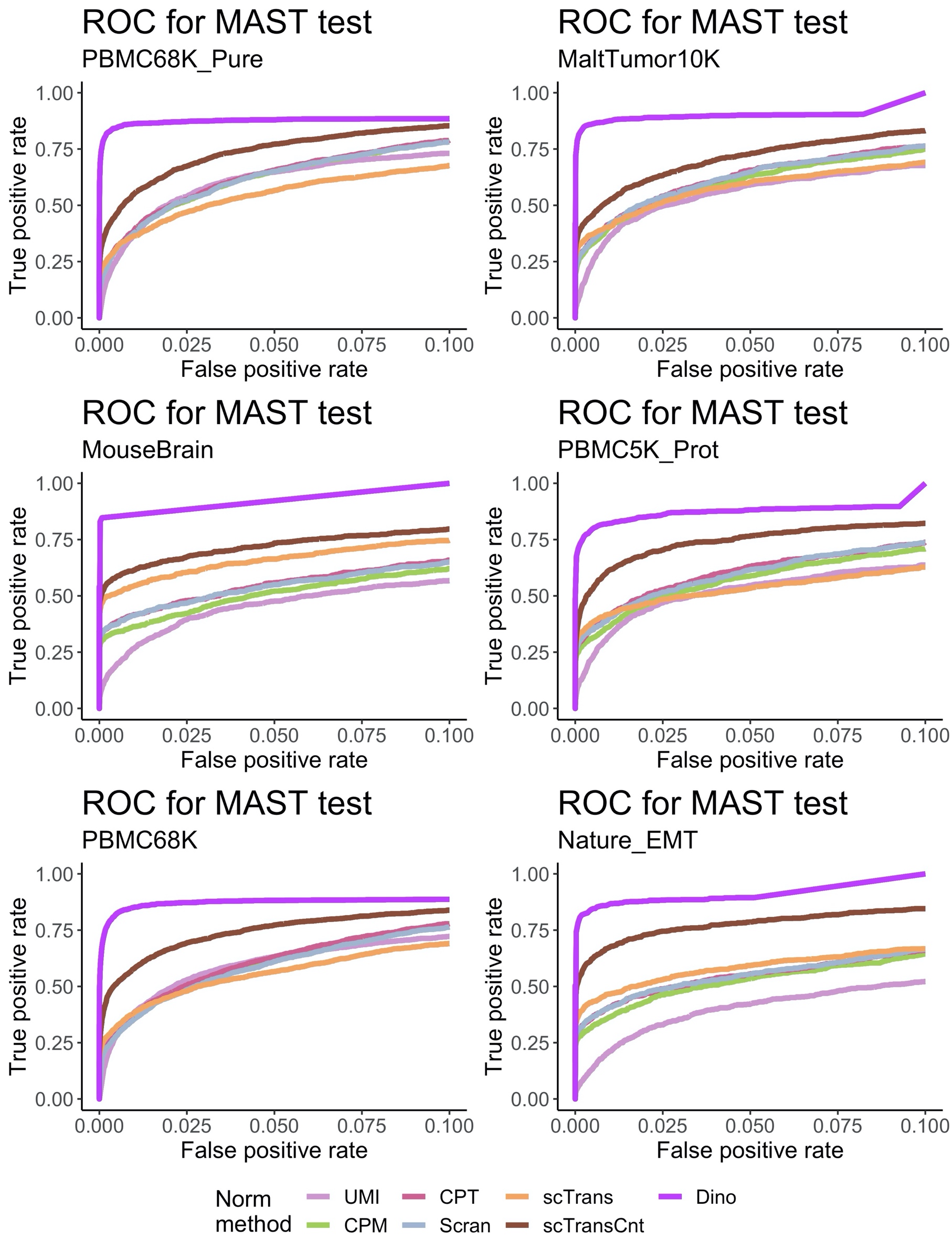


Supplemental Figure S17: **The effects of normalization on downstream DE analysis.** Simulated data based on each of the considered datasets were normalized using each method. ROC curves colored by normalization method define the relationship between average TPR (Power) and average FPR for a MAST test, where the average is calculated across 30 simulations from each dataset.


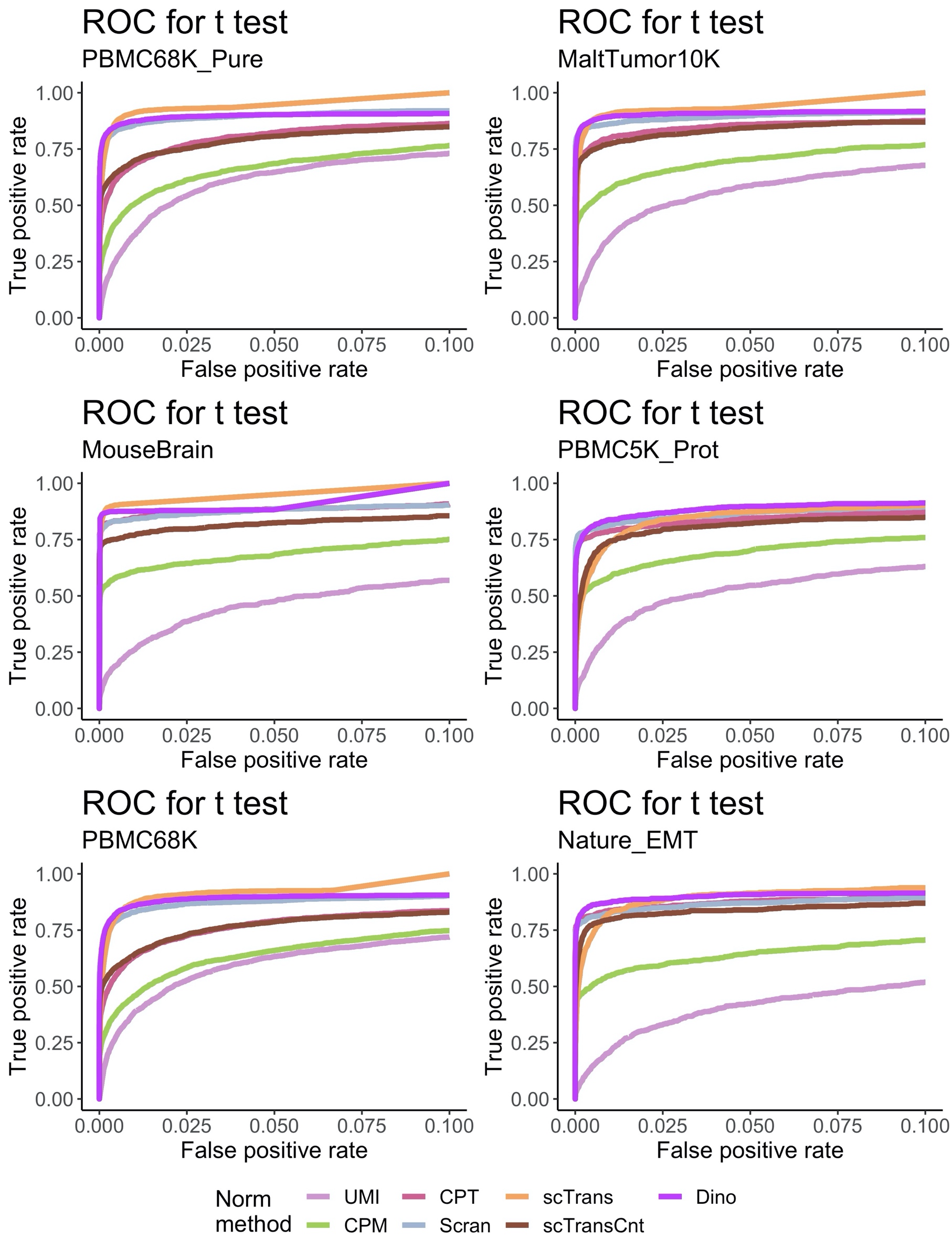


Supplemental Figure S18: **The effects of normalization on downstream DE analysis.** Simulated data based on each of the considered datasets were normalized using each method. ROC curves colored by normalization method define the relationship between average TPR (Power) and average FPR for a t-test, where the average is calculated across 30 simulations from each dataset.


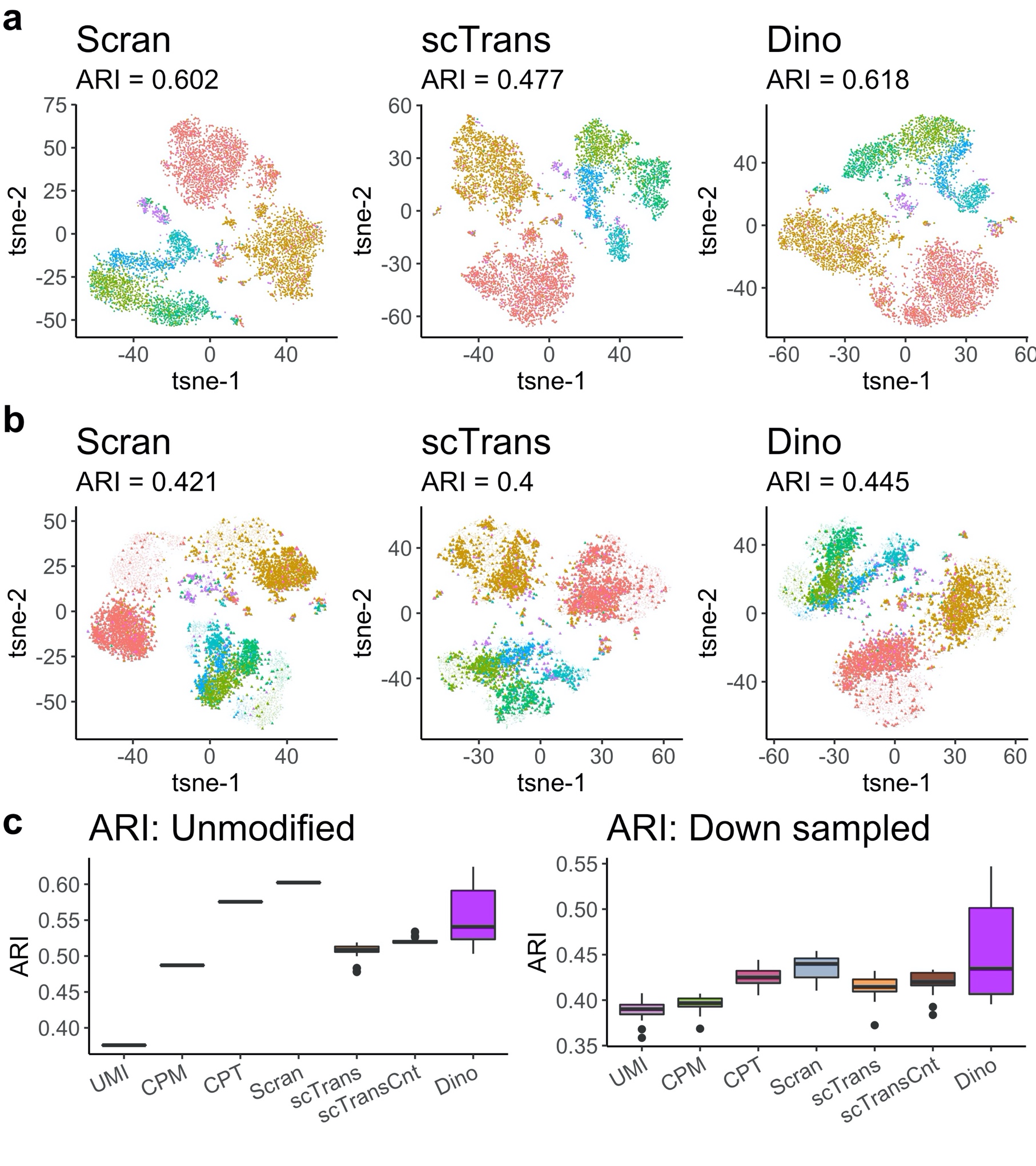


Supplemental Figure S19: **The effects of normalization on clustering.** a) tSNE plots of normalized MaltTumor10K data, colored by 11 pseudo-annotations, show similarly high accuracy across methods. b) The same clustering plots as in (a), but with half the data down-sampled prior to normalization to produce greater differences in LS. c) Boxplots of ARIs for multiple un-modified and down-sampled datasets across 24 replications of the normalization procedures and, for the down-sampled data, 24 applications of the down-sampling.


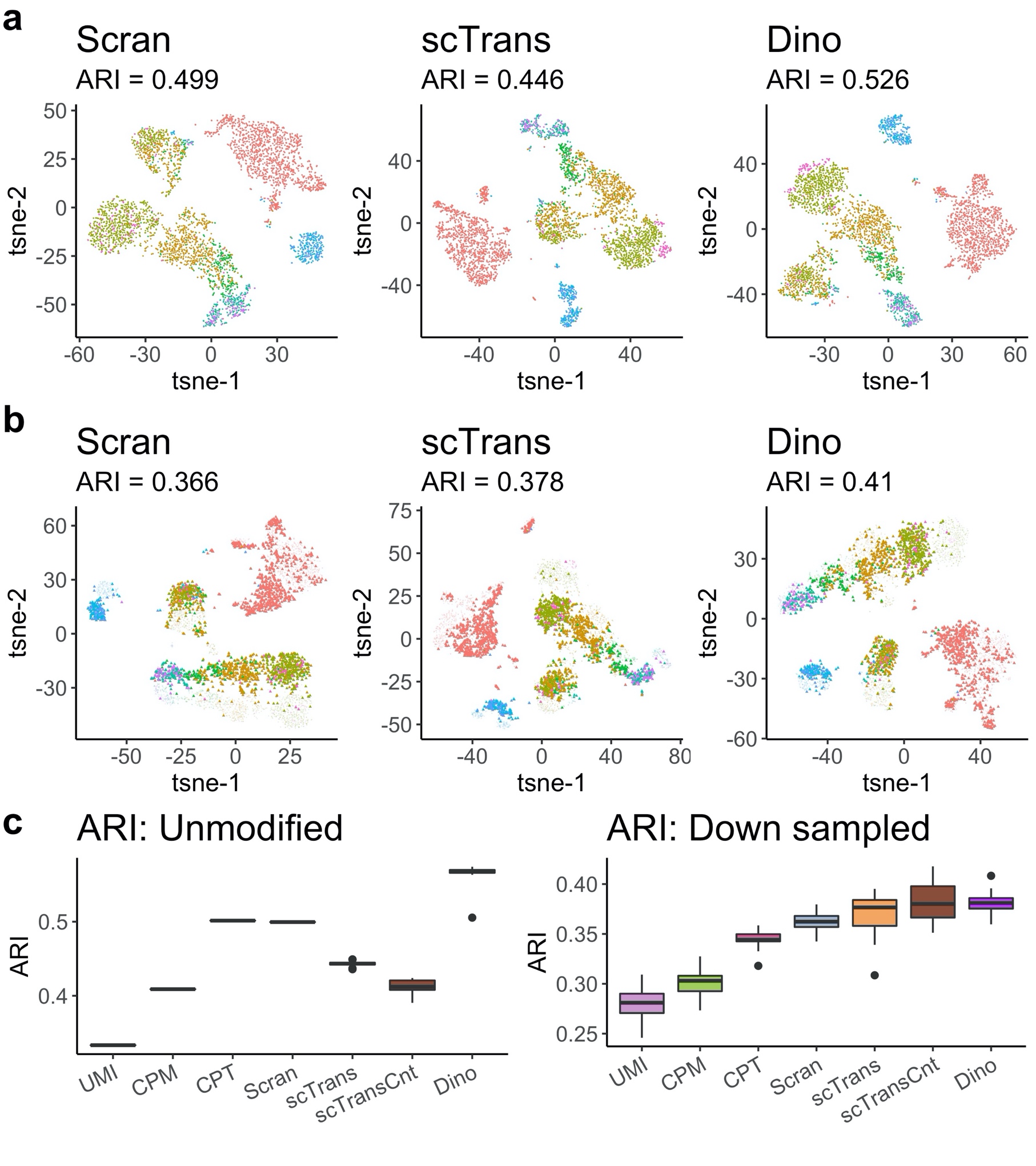


Supplemental Figure S20: **The effects of normalization on clustering.** a) tSNE plots of normalized PBMC5K_Prot data, colored by 11 pseudo-annotations, show similarly high accuracy across methods. b) The same clustering plots as in (a), but with half the data down-sampled prior to normalization to produce greater differences in LS. c) Boxplots of ARIs for multiple un-modified and down-sampled datasets across 24 replications of the normalization procedures and, for the down-sampled data, 24 applications of the down-sampling.


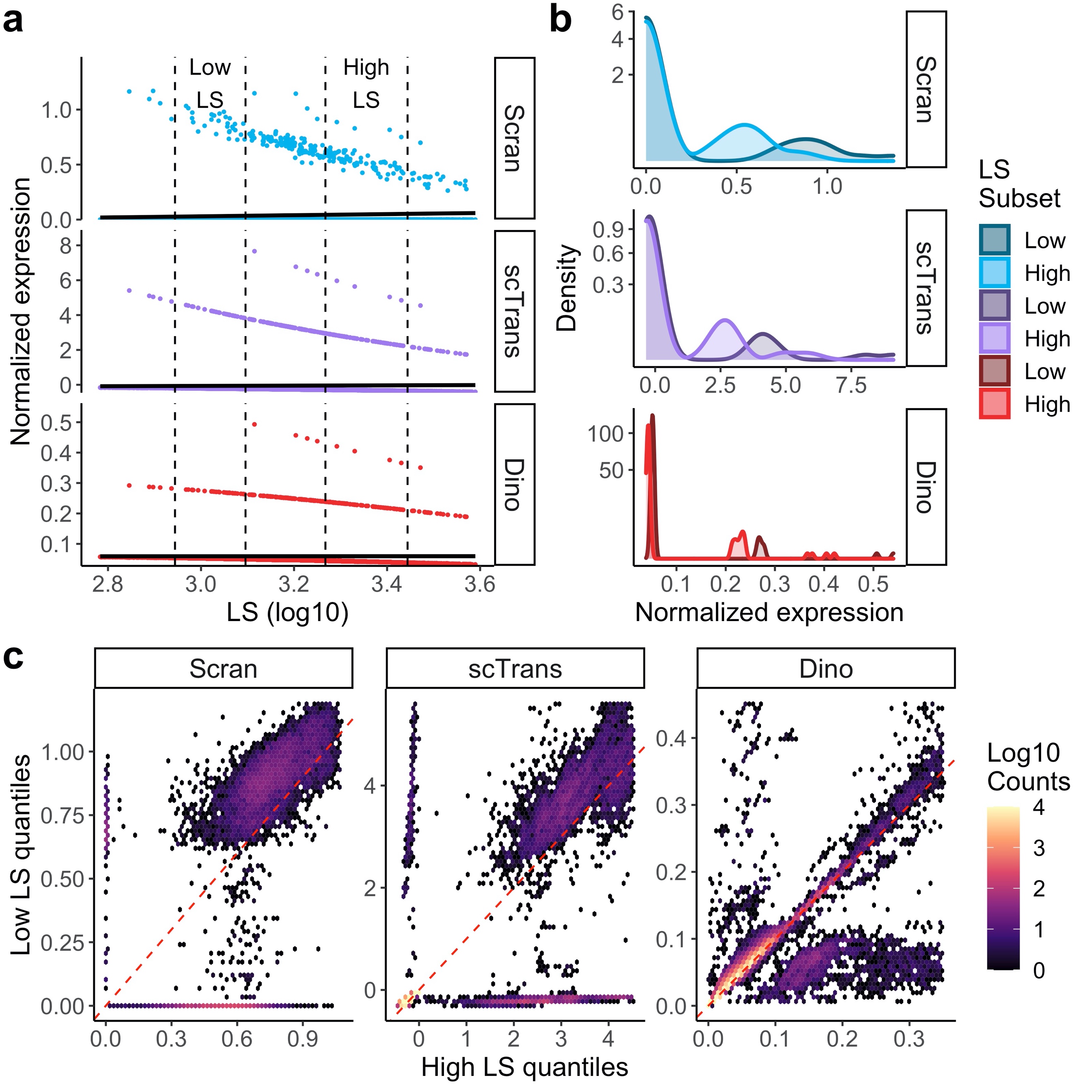


Supplemental Figure 21: **The effect of sampling from the posterior mean**. We repeat the analysis used to generate Fig 1 but substitute the default resampling procedure in Dino with one where normalized expression is estimated as the mean of the posterior distribution. In this way, it is observed that, absent resampling, the LS-dependent properties of the normalized expression distribution remain.

Supplemental Table S1: **Average power and FPR across simulated datasets and normalization methods.** 30 simulated datasets are produced from each case study dataset. In each simulation, the data are normalized by the panel of methods and significantly DE genes are identified using the Wilcoxon rank sum test. DE genes are defined as those with a Benjamini and Hochberg adjusted p-value less than 0.01. Average power and FPR is calculated over the 30 simulated datasets (standard error computed across simulations).

| **Norm. Method** | ***UMI*** | ***CPM*** | ***CPT*** | ***Scran*** | ***scTrans*** | ***scTransCnt*** | ***Dino*** |  |
| --- | --- | --- | --- | --- | --- | --- | --- | --- |
| **PBMC68K_Pure** | 0.797 (0.008) | 0.871 (0.006) | 0.871 (0.006) | 0.868 (0.006) | 0.894 (0.007) | 0.859 (0.008) | 0.818 (0.010) | **Power** |
|  | 0.196 (0.009) | 0.153 (0.010) | 0.153 (0.010) | 0.152 (0.010) | 0.622 (0.003) | 0.152 (0.008) | 0.007 (0.001) | **FPR** |
| **PBMC5K_Prot** | 0.738 (0.014) | 0.796 (0.013) | 0.796 (0.013) | 0.789 (0.013) | 0.838 (0.014) | 0.764 (0.014) | 0.729 (0.017) | **Power** |
|  | 0.244 (0.016) | 0.068 (0.014) | 0.068 (0.014) | 0.068 (0.014) | 0.630 (0.007) | 0.025 (0.005) | 0.004 (0.001) | **FPR** |
| **MaltTumor10K** | 0.774 (0.011) | 0.853 (0.009) | 0.853 (0.009) | 0.854 (0.009) | 0.923 (0.008) | 0.854 (0.009) | 0.808 (0.012) | **Power** |
|  | 0.216 (0.013) | 0.121 (0.013) | 0.121 (0.013) | 0.120 (0.013) | 0.704 (0.003) | 0.107 (0.010) | 0.003 (0.001) | **FPR** |
| **MouseBrain** | 0.788 (0.014) | 0.818 (0.011) | 0.818 (0.011) | 0.820 (0.011) | 0.896 (0.009) | 0.831 (0.009) | 0.751 (0.012) | **Power** |
|  | 0.440 (0.021) | 0.069 (0.014) | 0.069 (0.014) | 0.068 (0.014) | 0.516 (0.003) | 0.095 (0.013) | 0.000 (0.000) | **FPR** |
| **PBMC68K** | 0.799 (0.009) | 0.859 (0.006) | 0.859 (0.006) | 0.852 (0.007) | 0.896 (0.007) | 0.856 (0.007) | 0.823 (0.007) | **Power** |
|  | 0.202 (0.007) | 0.174 (0.008) | 0.174 (0.008) | 0.173 (0.008) | 0.595 (0.003) | 0.139 (0.012) | 0.009 (0.001) | **FPR** |
| **EMT** | 0.736 (0.013) | 0.812 (0.014) | 0.812 (0.014) | 0.812 (0.014) | 0.883 (0.014) | 0.824 (0.011) | 0.753 (0.015) | **Power** |
|  | 0.384 (0.017) | 0.092 (0.012) | 0.092 (0.012) | 0.094 (0.012) | 0.562 (0.007) | 0.040 (0.007) | 0.001 (0.000) | **FPR** |

Supplemental Table S2: **Average power and FPR across simulated datasets and normalization methods.** 30 simulated datasets are produced from each case study dataset. In each simulation, the data are normalized by the panel of methods and significantly DE genes are identified using the MAST test. DE genes are defined as those with a Benjamini and Hochberg adjusted p-value less than 0.01. Average power and FPR is calculated over the 30 simulated datasets (standard error computed across simulations).

| **Norm. Method** | ***UMI*** | ***CPM*** | ***CPT*** | ***Scran*** | ***scTrans*** | ***scTransCnt*** | ***Dino*** |  |
| --- | --- | --- | --- | --- | --- | --- | --- | --- |
| **PBMC68K_Pure** | 0.788 (0.008) | 0.956 (0.006) | 0.956 (0.006) | 0.963 (0.004) | 0.917 (0.006) | 0.906 (0.007) | 0.819 (0.010) | **Power** |
|  | 0.172 (0.008) | 0.274 (0.006) | 0.270 (0.005) | 0.299 (0.005) | 0.427 (0.012) | 0.179 (0.011) | 0.002 (0.000) | **FPR** |
| **PBMC5K_Prot** | 0.720 (0.014) | 0.943 (0.009) | 0.950 (0.009) | 0.966 (0.007) | 0.843 (0.011) | 0.799 (0.012) | 0.730 (0.016) | **Power** |
|  | 0.213 (0.015) | 0.346 (0.018) | 0.345 (0.016) | 0.372 (0.016) | 0.386 (0.021) | 0.071 (0.009) | 0.001 (0.000) | **FPR** |
| **MaltTumor10K** | 0.758 (0.010) | 0.953 (0.008) | 0.953 (0.007) | 0.962 (0.007) | 0.897 (0.008) | 0.905 (0.009) | 0.804 (0.011) | **Power** |
|  | 0.188 (0.012) | 0.213 (0.012) | 0.305 (0.010) | 0.327 (0.010) | 0.372 (0.020) | 0.182 (0.016) | 0.001 (0.000) | **FPR** |
| **MouseBrain** | 0.769 (0.015) | 0.962 (0.007) | 0.972 (0.007) | 0.969 (0.007) | 0.881 (0.010) | 0.880 (0.010) | 0.754 (0.012) | **Power** |
|  | 0.400 (0.020) | 0.521 (0.022) | 0.571 (0.021) | 0.561 (0.021) | 0.255 (0.021) | 0.208 (0.022) | 0.000 (0.000) | **FPR** |
| **PBMC68K** | 0.789 (0.008) | 0.946 (0.007) | 0.946 (0.006) | 0.963 (0.005) | 0.914 (0.008) | 0.870 (0.009) | 0.824 (0.007) | **Power** |
|  | 0.180 (0.006) | 0.268 (0.005) | 0.267 (0.004) | 0.304 (0.004) | 0.392 (0.007) | 0.144 (0.012) | 0.005 (0.000) | **FPR** |
| **EMT** | 0.715 (0.013) | 0.951 (0.010) | 0.961 (0.009) | 0.955 (0.009) | 0.889 (0.012) | 0.853 (0.011) | 0.744 (0.015) | **Power** |
|  | 0.349 (0.017) | 0.499 (0.012) | 0.508 (0.012) | 0.493 (0.012) | 0.349 (0.007) | 0.111 (0.007) | 0.000 (0.000) | **FPR** |

Supplemental Table S3: **Average power and FPR across simulated datasets and normalization methods.** 30 simulated datasets are produced from each case study dataset. In each simulation, the data are normalized by the panel of methods and significantly DE genes are identified using the t-test. DE genes are defined as those with a Benjamini and Hochberg adjusted p-value less than 0.01. Average power and FPR is calculated over the 30 simulated datasets (standard error computed across simulations).

| **Norm. Method** | ***UMI*** | ***CPM*** | ***CPT*** | ***Scran*** | ***scTrans*** | ***scTransCnt*** | ***Dino*** |  |
| --- | --- | --- | --- | --- | --- | --- | --- | --- |
| **PBMC68K_Pure** | 0.802 (0.008) | 0.810 (0.007) | 0.853 (0.007) | 0.868 (0.007) | 0.864 (0.009) | 0.864 (0.008) | 0.827 (0.010) | **Power** |
|  | 0.200 (0.009) | 0.149 (0.008) | 0.080 (0.006) | 0.014 (0.002) | 0.004 (0.000) | 0.130 (0.007) | 0.003 (0.000) | **FPR** |
| **PBMC5K_Prot** | 0.741 (0.014) | 0.776 (0.012) | 0.778 (0.013) | 0.776 (0.012) | 0.768 (0.013) | 0.772 (0.014) | 0.746 (0.014) | **Power** |
|  | 0.255 (0.016) | 0.120 (0.015) | 0.007 (0.002) | 0.001 (0.000) | 0.013 (0.001) | 0.017 (0.003) | 0.002 (0.000) | **FPR** |
| **MaltTumor10K** | 0.769 (0.011) | 0.792 (0.010) | 0.838 (0.009) | 0.847 (0.010) | 0.846 (0.012) | 0.859 (0.009) | 0.823 (0.011) | **Power** |
|  | 0.224 (0.013) | 0.134 (0.012) | 0.029 (0.005) | 0.004 (0.001) | 0.003 (0.000) | 0.070 (0.007) | 0.001 (0.000) | **FPR** |
| **MouseBrain** | 0.790 (0.013) | 0.810 (0.010) | 0.809 (0.011) | 0.808 (0.011) | 0.809 (0.012) | 0.834 (0.011) | 0.772 (0.012) | **Power** |
|  | 0.455 (0.022) | 0.184 (0.019) | 0.001 (0.001) | 0.002 (0.001) | 0.001 (0.000) | 0.063 (0.010) | 0.000 (0.000) | **FPR** |
| **PBMC68K** | 0.800 (0.009) | 0.802 (0.008) | 0.835 (0.006) | 0.853 (0.007) | 0.861 (0.007) | 0.852 (0.007) | 0.837 (0.007) | **Power** |
|  | 0.206 (0.006) | 0.161 (0.006) | 0.096 (0.005) | 0.018 (0.002) | 0.008 (0.001) | 0.124 (0.012) | 0.006 (0.000) | **FPR** |
| **EMT** | 0.732 (0.013) | 0.765 (0.015) | 0.788 (0.015) | 0.782 (0.015) | 0.832 (0.010) | 0.823 (0.011) | 0.778 (0.013) | **Power** |
|  | 0.397 (0.017) | 0.175 (0.016) | 0.001 (0.000) | 0.002 (0.001) | 0.011 (0.001) | 0.024 (0.004) | 0.001 (0.000) | **FPR** |
